# *Peganum harmala*-mediated green synthesis of Ag, Cu, and Ag–Cu bimetallic nanoparticles for the chemical mitigation of nickel-induced oxidative stress in *Triticum aestivum* L.

**DOI:** 10.1039/d6ra03724k

**Published:** 2026-07-03

**Authors:** Amjid Khan, Tauqeer Ahmed Qadri, Rashid Abbas Khan, Muhammad Anas, Dilawar Hassan, Ayesha Sani, Bushra Ashiq, Zabta Khan Shinwari, Malik Maaza

**Affiliations:** a UNESCO-UNISA Africa Chair in Nanosciences and Nanotechnologies, College of Graduate Studies, University of South Africa 1 Preller Street, Muckleneuk Ridge, P. O. Box 392 Pretoria Gauteng Province 0003 South Africa khana2@unisa.ac.za; b African Centre of Competencies in Enhanced Nanosciences & Nanotechnologies for SDGs (ACCENTS) 1 Preller Street, Muckleneuk Ridge, P. O. Box 392 Pretoria Gauteng Province 0003 South Africa; c Department of Biosciences, COMSATS University Islamabad Islamabad 45550 Pakistan; d Department of Soil Science & Plant Nutrition, Selçuk University Campus Konya 42079 Turkey; e Department of Biomedical Engineering, Research Center for Nano-biomaterials & AMP; Regenerative Medicine, College of Artificial Intelligence, Taiyuan University of Technology Taiyuan 030024 P. R. China; f Department of Plant Sciences, Faculty of Biological Sciences, Quaid-i-Azam University Islamabad 45320 Pakistan shinwari@qau.edu.pk; g Federal Urdu University of Arts, Sciences and Technology (FUUAST) Karachi 75300 Pakistan

## Abstract

The remediation of heavy metal contamination in agricultural soils requires sustainable, eco-friendly agronomic interventions. This study reports the application of pre-synthesized and thoroughly pre-characterized silver (Ag), copper (Cu), and Ag–Cu bimetallic nanoparticles (NPs) originally fabricated *via* a *Peganum harmala*-mediated green synthesis pathway. We investigate these pre-characterized NPs as nano-chemical regulators to mitigate nickel (Ni) toxicity in wheat (*Triticum aestivum* L.), focusing on the relationship between nanoparticle composition (monometallic *vs.* bimetallic) and the modulation of the plant's chemical defense and metal translocation pathways. While the baseline structural baseline, including crystalline properties, chemical capping, and size distribution (20–30 nm), was established in our prior work *via* UV-Vis, XRD, FTIR, SEM/TEM, and DLS analyses, the current investigation focuses entirely on evaluating their downstream agronomic efficacy. Application of 250 mg L^−1^ NPs *via* seed priming and foliar spray under 100 mg kg^−1^ Ni stress significantly altered the plant's biochemical profile. Cu-NPs provided the most robust growth response, increasing grain yield by 21.3% in the Borlaug-16 cultivar. Chemically, the Ag–Cu bimetallic system demonstrated superior antioxidant stimulation, enhancing total phenolic and anthocyanin contents by 112.82% and 116.86%, respectively. This was accompanied by a synergistic enhancement of SOD (90.7%) and POD (122%) enzymatic activities, which led to an 82.7% reduction in H_2_O_2_ oxidative markers. Crucially, ionomic analysis revealed that Ag–Cu NPs act as chemical facilitators for Ni mobility, increasing the translocation factor from roots to grains (TF up to 4.2). These findings demonstrate that repurposing these green-synthesized nanoparticles effectively reprograms the wheat plant's chemical response to heavy metal stress. While the bimetallic Ag–Cu NPs provide superior biochemical protection against oxidative damage, they simultaneously promote the mobilization of Ni into edible tissues. This study highlights a critical chemical trade-off between physiological resilience and toxicological safety, providing essential data for the design of “smart” nano-agrochemicals that balance crop productivity with food safety requirements.

## Abstract 2

This study evaluates the potential of *Peganum harmala* extract, Ag-NPs, Cu-NPs, and bimetallic Ag–Cu NPs to alleviate nickel stress, reduce heavy metal translocation, and enhance growth and yield metrics in two wheat cultivars.

## Graphical abstract

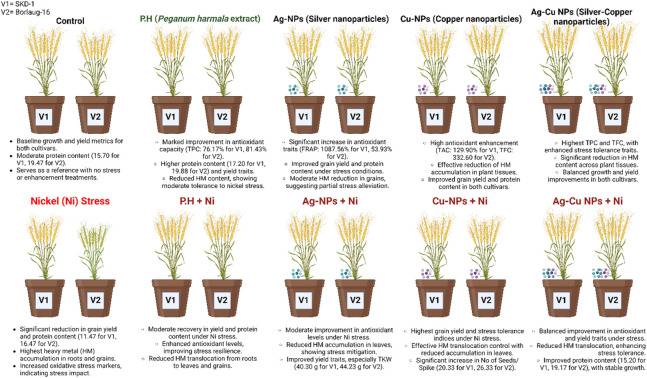

## 1. Introduction

Heavy metal pollution in agricultural soils is a pressing global issue, significantly impacting crop health, productivity, and food safety.^1^ Addressing these escalating environmental challenges requires highly efficient mitigation strategies and the development of advanced functional materials. Recent literature highlights the transformative potential of engineered nanomaterials, functionalized composites, and green polymers across diverse applications, ranging from comprehensive environmental remediation and water purification,^2,3^ to advanced degradable polymers,^4^ ultrasensitive electrochemical sensing platforms,^5^ and the structural engineering of robust energy materials.^6^ Inspired by these cross-disciplinary advancements in nanotechnology, researchers are increasingly applying functional nanomaterials to agricultural settings. Heavy metals such as cadmium, chromium, lead, and nickel persist in soils due to their non-biodegradable nature and tend to accumulate to levels that impair essential crop functions, affecting growth, nutrient uptake, and metabolism.^7^ Recent research highlights that even essential micronutrients, when present in toxic concentrations, can disrupt the antioxidant balance and hormonal signaling within the plant, further complicating the physiological response to soil contamination.^1^ Agricultural systems are often contaminated with nickel (Ni) from many sources, including discharges of industry, mining, sewage sludge, and the use of phosphate fertilizers and pesticides.^8,9^ Not only was there significant disruption to metabolic pathways and oxidative stress in crops in general, but specifically, these free ions are known, disrupt wheat at high levels, causing structural and physiological changes, including ion imbalance and cellular damage. The effects of nickel on wheat cultivars SKD-1 and Borlaug-16 at the vegetative stage, having disturbed agronomic, morphological and physiological changes, have been recently studied.^3^ Despite these preliminary insights, a critical knowledge gap remains regarding the comparative efficacy of green-synthesized monometallic *versus* bimetallic nanoparticles in managing Ni toxicity across the full wheat life cycle, particularly regarding grain yield and tissue-specific Ni partition. There is currently limited data on how the synergy between Ag and Cu in a bimetallic framework influences Ni translocation and the long-term recovery of wheat agronomic traits.

Reactive oxygen species (ROS) are generated in nickel-stressed cells in the form of superoxide radicals and hydrogen peroxide, resulting in oxidative stress that damages cellular components, including lipids, proteins, and DNA.^3,10^ The key physiological processes, such as photosynthesis, nutrient uptake, and membrane stability, are impaired in plants due to this oxidative imbalance. In response to these effects, plants must robustly defend against oxidative damage utilizing both enzymatic (superoxide dismutase (SOD), catalase (CAT), and peroxidase (POD)) and non-enzymatic compounds (phenolics, flavonoids, AsA), which act as antioxidants.^11–13^ Enzymatic antioxidants play specific roles:^14^ report that SOD converts superoxide radicals to less harmful molecules, and CAT and POD detoxify hydrogen peroxide to water and oxygen, respectively, to prevent cellular damage. At the same time, phenolics and flavonoids are free radical scavengers and can stabilize the redox balance.^15,16^ In addition, AsA provides additional protection as it can neutralize ROS and regenerate other antioxidants, which will maintain cellular integrity under Ni stress.^17^ Together, the metabolic and antioxidant response to Ni enhances the plant's resistance to Ni, allowing the plant to survive and grow, and produce without cellular dysfunction in metal-polluted environments.^18^

However, remediation of Ni-contaminated soils is necessary to sustain crop health and yield, because overexposure of nickel inhibits plant growth, disturbs uptake of essential nutrients, and impairs food quality and safety.^19^ However, conventional remediation methods, such as soil washing and phytoremediation, are frequently inefficient and demand long-term management, which has therefore stimulated the development of new solutions.^20^ The enhanced reactivity and bioavailability of bimetallic nanoparticles have made them a promising approach for mitigating heavy metal toxicity.^21–23^ Bimetallic nanoparticles, such as Ag–Cu, are hypothesized to provide superior stress mitigation compared to their single-metal counterparts due to synergistic electronic effects and increased surface area-to-volume ratios. Mechanistically, the combination of Ag's ability to modulate ethylene signaling and Cu's role as a vital cofactor for antioxidant enzymes (like Cu/Zn-SOD) may offer a dual-action defense system that more effectively suppresses ROS and regulates metal uptake than either metal used alone. Green-synthesized nanoparticles offer an eco-friendly, sustainable, and cost-effective alternative to conventional methods by eliminating toxic chemicals, reducing environmental impact, enhancing biocompatibility, improving functional properties, and supporting applications in agriculture and environmental remediation.^24–26^ Nanoparticles, due to their enhanced surface reactivity and bioavailability, offer a promising approach for mitigating heavy metal toxicity by improving plant antioxidant defense, enhancing nutrient uptake, and reducing metal accumulation, thereby promoting growth and resilience in contaminated environments.^27,28^

We hypothesize that Ag, Cu, and Ag–Cu NPs will enhance wheat tolerance to nickel stress by improving growth, antioxidant defense, and minimizing nickel accumulation. This study explores both nano-seed priming and foliar application of single-metal (Ag, Cu) and bimetallic (Ag–Cu) nanoparticles, creating a multi-faceted approach to nickel stress mitigation. The research aims to assess Ag, Cu, and Ag–Cu nanoparticles applications on wheat's agronomic, phytochemical, physiological, and biochemical responses, focusing on chlorophyll content, growth stages, yield, and antioxidant profiles, providing a deeper understanding of how these nanoparticles mitigate stress effects and promote plant resilience.

## 2. Materials and methods

### 2.1. Experimental framework and plant material

This experiment was done at Quaid-i-Azam University, Islamabad, Pakistan with application of Ni stress in a controlled glasshouse in which light, temperature, and humidity were optimized in such a way that the photographed plants could withstand external application of stress. From the same laboratory, wheat seeds of cultivars SKD-1 (V1) and Borlaug-16 (V2) were obtained.^29^ also identified V1 for general tolerance to heavy metals, and V2 for specific resistance to nickel stress, applied across treatments as 100 mg kg^−1^ Ni stress (NiCl_2_). While 100 mg kg^−1^ Ni represents levels found in heavily industrialized and mining-impacted areas, this concentration was specifically chosen to simulate an “upper-limit” agronomic stress scenario. This ensures that the mitigative effects of the nanoparticles are tested against a robust toxicological challenge, providing clear results on their potential for restoring crop health in contaminated marginal lands.^30^

Ag, copper Cu, and Ag–Cu bimetallic NPs were synthesized using *P*. *harmala* extract as a green reducing and stabilizing agent, following a modified method from ref. 31. The *P*. *harmala* plant material was collected in June 2023 from Village Sawans, District Mianwali, Punjab Province, Pakistan, with explicit permission from the landowner. The taxonomic identification of the plant was carried out at the Department of Plant Sciences, Quaid-i-Azam University (QAU), Islamabad, by Prof. Dr Zabta Khan Shinwari, an expert taxonomist. A voucher specimen (no. 133568) has been deposited in the Herbarium of Pakistan (ISL) for documentation and future reference. A 1 mM solution of AgNO_3_ and CuSO_4_ was mixed with the extract under controlled stirring at 60 °C, leading to nanoparticle formation, confirmed by color changes. A 1 mM solution of AgNO_3_ and CuSO_4_ was mixed with the extract under controlled stirring at 60 °C, leading to nanoparticle formation, confirmed by color changes. The nanoparticles were purified *via* centrifugation (10 000 rpm, 20 min) and dried at 60 °C. The baseline structural, optical, and morphological characterization of these identical green-synthesized batches using UV-Vis, XRD, FTIR, SEM, TEM, EDS, and DLS has been comprehensively reported in our recently published work focusing on their biomedical and catalytic profiles.^32^ These prior analyses confirmed their highly crystalline nature, structural stability, elemental purity, and optimal size range (20–30 nm), establishing their physical suitability for the environmental and agronomic applications evaluated in the present study. Remediation strategies in this study involved the application of silver, copper, and Ag–Cu bimetallic NPs synthesized using *P. harmala* extract. These nanoparticles were applied using a dual approach of nano-seed priming and foliar spraying at a concentration of 250 ppm. As detailed in ref. 32, the pre-characterized particles exhibited distinctive physicochemical signatures: Ag nanoparticles exhibited a surface plasmon resonance (SPR) peak at 425 nm, Cu-NPs at 555 nm, and Ag–Cu bimetallic NPs at 525 nm. Crystallite sizes, determined through X-ray diffraction, were 21.42 nm for Ag-NPs, 21.40 nm for Cu-NPs, and 26.29 nm for Ag–Cu bimetallic NPs. Morphological analysis revealed that Ag-NPs had a spherical shape, Cu-NPs exhibited a flake-like structure, Ag–Cu bimetallic NPs showed a combination of cubic and spherical shapes.^38^ This simultaneous application strategy was designed to provide comprehensive protection throughout the plant life cycle: seed priming addresses early germination-stage sensitivity, while foliar application targets photosynthetic machinery and reproductive development. While we acknowledge that this integrated approach does not isolate the individual contribution of each method, it was chosen to maximize the synergistic potential for stress mitigation in a multi-stage growth study.^22,33^ This dual approach is to enhance the plants' defense against Ni-induced oxidative damage through antioxidant systems during the whole development cycle, and to provide a thorough evaluation of the nanoparticles' effect under the controlled Ni stress conditions.

The experimental treatments included a control group with no exposure to stress or nanoparticles, and a group exposed to Ni stress alone (Ni). Another treatment involved *P. harmala* extract at a concentration of 250 ppm without nanoparticles (P.H). Additional treatments included *P. harmala* extract combined with Ni stress (P.H + Ni) and nanoparticle treatments using a 250 ppm solution of nanoparticles: silver nanoparticles (Ag-NPs), copper nanoparticles (Cu-NPs), and silver–copper bimetallic nanoparticles (Ag–Cu NPs). Further combinations were tested, including Ag-NPs with Ni stress (Ag-NPs + Ni), Cu-NPs with Ni stress (Cu-NPs + Ni), and Cu–Ag bimetallic NPs with Ni stress (Ag–Cu NPs + Ni). This setup was designed to strengthen the antioxidant systems and resilience of wheat plants throughout the growth cycle, providing comprehensive insights into the potential of nanoparticles to mitigate Ni-induced stress.

### 2.2. Soil analysis methodology

The study followed a completely randomized design (CRD) with a factorial arrangement. Each treatment was replicated three times (3 biological replicates). Each experimental unit consisted of a 3 kg plastic pot containing four wheat plants. Pots were repositioned weekly to ensure uniform exposure to glasshouse conditions. Air-dried soil samples were taken from a designated site and sieved through a 2 mm mesh to remove debris. A colorimetric method employing nitrate reagents was utilized to determine the nitrate content, in which Absorbance was used to quantify nitrate concentration in the mg kg^−1^ nitrogen unit. Soil pH was measured at 1 : 2. Once calibrated, a pH meter should be used to read accurately soil acidity or alkalinity in 5 suspensions, soil to water. The same suspension was used to determine the electrical conductivity (EC) using an EC meter, and such data on soil salinity.^34^

Oxidizable carbon was measured according to the Walkley–Black method, expressed as a percentage of the soil sample for organic carbon analysis. Oxidizable carbon measurements were used to calculate total organic carbon (TOC) and organic matter (OM). The Olsen method was used to quantify phosphates in the soil sample, where the extracting solution, which was added to the soil sample, then had phosphate levels measured using spectrophotometric analysis at a defined wavelength. Altogether, these procedures yield a complete chemical profile of the soil that is required for determining its utility for agricultural applications.^34,35^

### 2.3. Growth and agronomic analysis

For assessing growth and agronomic traits in wheat under nickel stress, we tracked several morphological, physiological, and yield-related parameters across the crop's development stages. Growth stages, including sowing, germination, tillering, booting, heading, and anthesis, were recorded to monitor plant progression accurately. Key metrics such as days to germination (DTG), days to tillering (DTT), days to booting (DTB), days to heading (DTH), and days to anthesis (DTA) were noted to gauge developmental benchmarks under stress. Chlorophyll content was measured at each major growth stage: tillering, booting, heading, and anthesis, using a SPAD chlorophyll meter to evaluate photosynthetic efficiency, as chlorophyll levels correlate strongly with plant health and stress response. Biomass and structural adaptation to stress were also assessed by measurement of physiological traits, such as fresh weight, leaf length, width, and leaf area (fLA) at the heading and anthesis stages. Insights were gained into yield potential under conditions of stress by agronomic traits of number of seeds per spike, plant height, spike length, tillers per plant, seeds per spikelet, biological yield (BY), grain yield, thousand kernel weight (TKW) as per ref. 36.

### 2.4. Analysis of Ni uptake, accumulation, and translocation to above-ground parts of wheat

We quantified Ni uptake, accumulation, and translocation in wheat with the analysis of Ni in soil (HM-soil), root (HM-root), leaf (HM-leaf), and grain (HM-grain). To understand Ni movement through the plant, we calculated translocation factors (TF) of root to leaf (TF-root/leaf) and of leaf to grain (TF-leaf/grain). Both bioconcentration factors (BCF-soil/root) and bioaccumulation factor (BAF) were used to compare Ni accumulation in roots and leaves and soil to assess uptake efficiency and translocation potential. They allow that to determine dynamics in Ni within the plant, necessary for evaluating tolerance and remediation strategies.^37,38^

1 TF1 = Ni_leaf_/Ni_root_

2 TF2 = Ni_grain_/Ni_leaf_

3 BCF = Ni_root_/Ni_soil_

4 BAF = Ni_leaf_/Ni_soil_

### 2.5. Phytochemical content analysis of leaf

Total phenolic content (TPC) was measured using the Folin–Ciocalteu method, with results expressed as gallic acid equivalents (GAE). This assay quantifies phenolic compounds by their reaction with the Folin–Ciocalteu reagent, producing a color change that is measured spectrophotometrically at 760 nm.^39^ Total flavonoid content (TFC) was assessed through the aluminum chloride colorimetric method, using quercetin as a standard, with results expressed as quercetin equivalents (QE). The method involves the formation of a complex between aluminum and flavonoids, yielding an absorbance at 510 nm.^40^ Total anthocyanin content (TAnC) was measured using the pH differential method by recording absorbance at 520 nm and 700 nm in buffers at pH 1.0 and 4.5. The absorbance difference was calculated and used to quantify TAnC as cyanidin-3-glucoside equivalents.^41^ Total tannin content was determined using the Folin–Ciocalteu method. Absorbance at 765 nm measured phenolic compounds, and tannins were quantified by treating the sample with a protein precipitant to remove tannins. The difference in absorbance was expressed as tannic acid equivalents.^42^ Ascorbic acid content was determined using the 2,4-dinitrophenylhydrazine (DNPH) method. In this procedure, ascorbic acid is oxidized to dehydroascorbic acid, which reacts with DNPH to form a hydrazone derivative. This derivative, upon treatment with sulfuric acid, yields a colored complex whose absorbance is measured spectrophotometrically at 520 nm. The intensity of the color is directly proportional to the ascorbic acid concentration, allowing for its quantification.^43^

### 2.6. Antioxidant capacity and activity

Multiple assays were used to analyze total antioxidant capacity. The green phosphate Mo(v) complex was produced by the phosphomolybdenum method, which measured the reduction of Mo(vi) to Mo(v) at 695 nm.^44^ The reduction of Fe^3+^ to Fe^2+^ was quantified by the ferric reducing antioxidant power (FRAP) assay, producing the blue Fe^2+^–TPTZ complex at 593 nm.^45^ Neutralization of the DPPH radical by antioxidants was estimated from the decrease in absorbance at 517 nm, with respect to the 2,2-diphenyl-1-picrylhydrazyl (DPPH) radical scavenging assay.^46^ Quenching of ABTS^+^ radical cations by the 2,2′-azino-bis(3-ethylbenzothiazoline 6-sulfonic acid) (ABTS) assay was measured at 734 nm using both hydrophilic as well as lipophilic antioxidants.^47^

#### 2.6.1. Enzymatic antioxidants

Spectrophotometric quantification of enzymatic antioxidant activities was accomplished. Superoxide dismutase (SOD; EC 1.15.1.1) was determined by monitoring absorbance at 560 nm as previously described, with activity expressed in units per gram of fresh weight (U g_FW_^−1^). POD (EC 1.11.1.7) activity was measured at 420 nm, using U g_FW_^−1^. Absorbance at 240 nm was recorded based on catalase (CAT; EC 1.11.1.6) activity and expressed as U min^−1^ g_FW_^−1^ using the method of ref. 48 for all three enzymes.

#### 2.6.2. Oxidative stress indicators

A spectrophotometric method was used to measure the levels of hydrogen peroxide (H_2_O_2_) in leaves and roots using absorbance at 390 nm. The lipid peroxidation was assessed based on malondialdehyde (MDA) content by the thiobarbituric acid (TBA) method. Specific measurements were done at 532 nm, while nonspecific absorbance was 600 nm, and results were expressed as mmol g^−1^ fresh weight.^49^

### 2.7. Grain nutrition assessment

TPC, TFC, and TTC were quantified using Folin–Ciocalteu, aluminum chloride colorimetric, and vanillin–HCl methods according to the procedure described in the previous section. Assessments of non-reducing sugars (NRS), reducing sugars (RS), total sugars (TS), and starch content after ref. 38 were included in the carbohydrate profile. Carbohydrate analysis used spectrophotometric methods, primarily *via* a standard curve calibration, as is commonly done in cereal grain analysis to provide a reliable measurement of different sugar types and starch as the major energy sources. Protein was determined by Kjeldahl or Dumas, both of which are widely used in food science, and measure nitrogen content in grains, and calculate protein content.^50^

### 2.8. Statistical analysis

Statistical analysis data were analyzed using a three-way factorial analysis of variance (ANOVA) to determine the main effects and the significance of interactions between the three factors: wheat cultivar (V), Ni, and nanoparticle treatment (T). R Studio 4.3.3 and Origin 2024 were used for statistical analysis. *Post hoc* comparisons were conducted using Tukey's HSD test (or Bonferroni corrections where applicable) at a significant level of *p* < 0.05 to identify specific differences between group means. Treatment effects are illustrated through data visualization (bar graphs), and for non-normal data distributions, appropriate non-parametric alternatives were utilized.

## 3. Results

### 3.1. Analysis of morpho-agronomic traits and stress tolerance indices

**Fig. 1:**
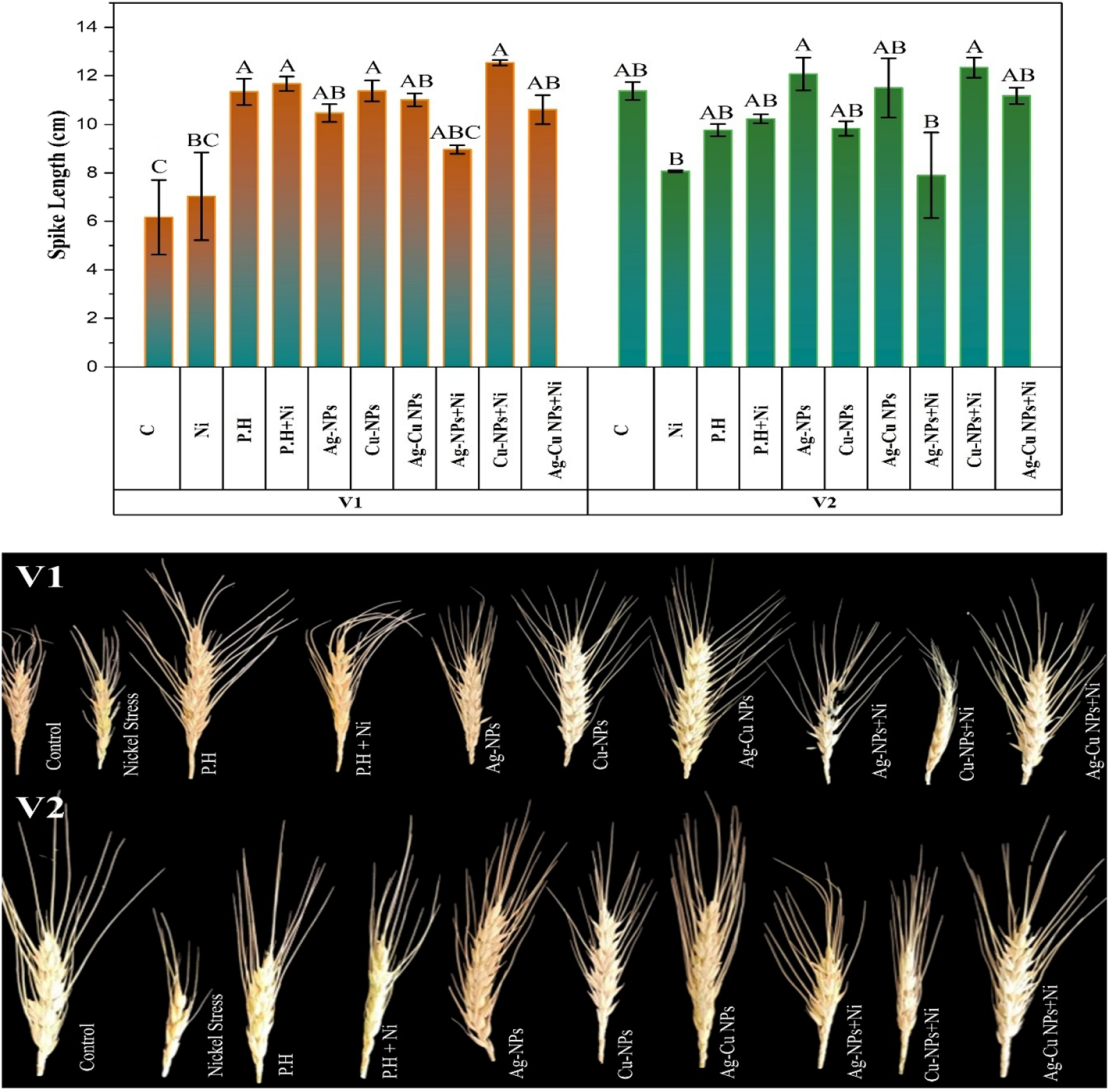
Spike length (cm) of wheat cultivars V1 and V2 under different treatments with and without Ni stress. The first panel shows spike morphology across treatments, while the second panel quantifies spike length, with letters indicating significant differences (*p* < 0.05). Cu-NPs and Ag–Cu NPs with Ni stress showed the highest spike lengths in both cultivars, indicating improved growth under stress. Error bars represent standard deviation.

Under nickel stress, significant variations were observed in plant height, leaf area, spike length, and biomass across treatments, alongside key agronomic traits like BY, GY, and TKW. The application of Cu-NPs showed pronounced improvements in growth for both V1 and V2 cultivars, with Cu-NPs yielding the highest plant height (22.33 cm for V1 and 20.17 cm for V2) and spike length (12.53 cm and 12.33 cm, respectively) under combined Ni stress conditions. BY was notably higher in Cu-NPs + Ni treatment for both cultivars, with V2 achieving the highest BY at 2.85 g per plant. This treatment also resulted in the highest GY for V2 (0.91 g), while V1 showed a GY of 0.75 g under the same treatment. Thousand kernel weight, a critical quality parameter, was optimized in V1 with Ni treatment alone (133.08 g) and in V2 with plant height (P.H) treatment (45.57 g), indicating improved grain weight under these conditions. Similarly, spike length was greatest in Cu-NPs + Ni treatments for V1 (12.53 cm) and V2 (12.33 cm), indicating superior growth and stress tolerance. Under various treatments, SpL exhibited cultivar-specific responses. For V1, the highest spike length was observed with P.H treatment at 13.50 cm, followed by P.H + Ni at 12.40 cm and Cu-NPs + Ni at 12.10 cm (Fig. 1). Ag–Cu NPs treatment achieved a moderate spike length of 10.10 cm, while the control and Ni treatments resulted in the shortest spikes at 9.00 cm. In V2, Ag–Cu NPs showed the greatest enhancement in spike length at 16.20 cm, followed closely by Ag-NPs at 14.10 cm and Cu-NPs + Ni at 14.00 cm. Ni alone had the lowest spike length at 8.20 cm. Combined nanoparticle treatments with Ni generally improved spike length, reflecting a trend of increased growth under certain nanoparticle treatments in both cultivars.

Chlorophyll content, measured at critical growth stages, illustrated improved photosynthetic efficiency in nanoparticle treatments, especially Cu-NPs and Ag–Cu NPs. At the anthesis stage, chlorophyll readings for V1 were highest in Cu-NPs treatment (42.63), while V2 showed elevated levels in Ag-NPs (37.81) and Cu-NPs treatments (43.82), suggesting a reduction in stress impact and increased photosynthetic resilience.

**Fig. 2:**
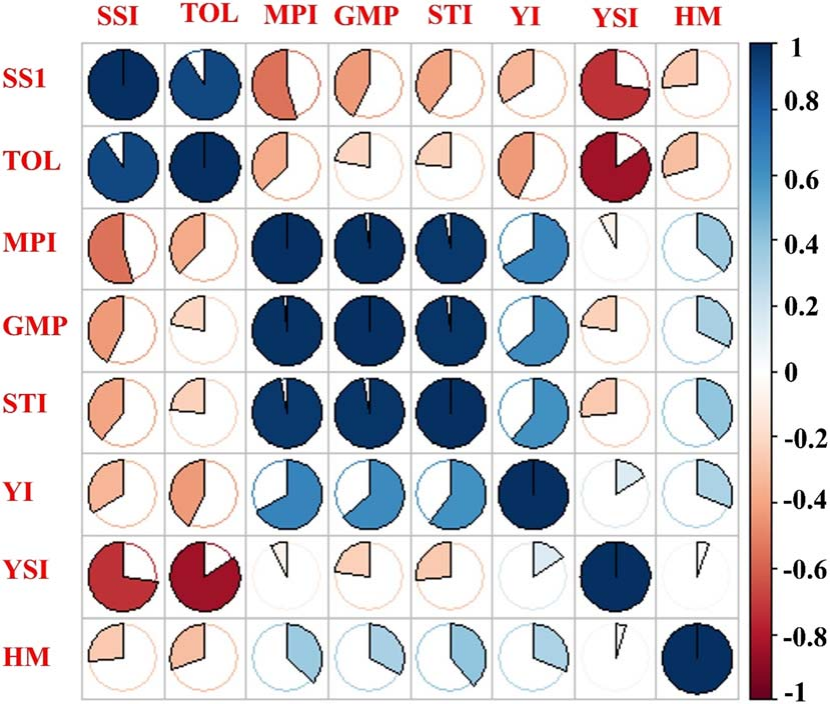
Correlation matrix of stress tolerance indices in wheat cultivars under nickel stress, represented through a pie-chart visualization. Each cell shows the correlation between two indices, with the color and size of the pie segments indicating the strength and direction of the correlation. Positive correlations are shown in shades of blue, and negative correlations in shades of red, with the intensity reflecting the correlation magnitude. Indices include susceptibility index (SSI), tolerance index (TOL), mean productivity index (MPI), geometric mean productivity (GMP), stress tolerance index (STI), yield index (YI), yield stability index (YSI), and harmonic mean (HM), highlighting their interrelationships and contributions to stress tolerance assessment.

Stress tolerance indices, including the TOL, MPI, GMP, and STI, were favorable under Cu-NPs treatments, indicating improved tolerance and resilience (Fig. 2). The SSI values were lowest for Ag–Cu NPs and Cu-NPs treatments, reflecting greater stress tolerance. YSI and HM further supported Cu-NPs as the most effective treatment, exhibiting robust growth and reduced sensitivity to nickel-induced stress.

**Fig. 3:**
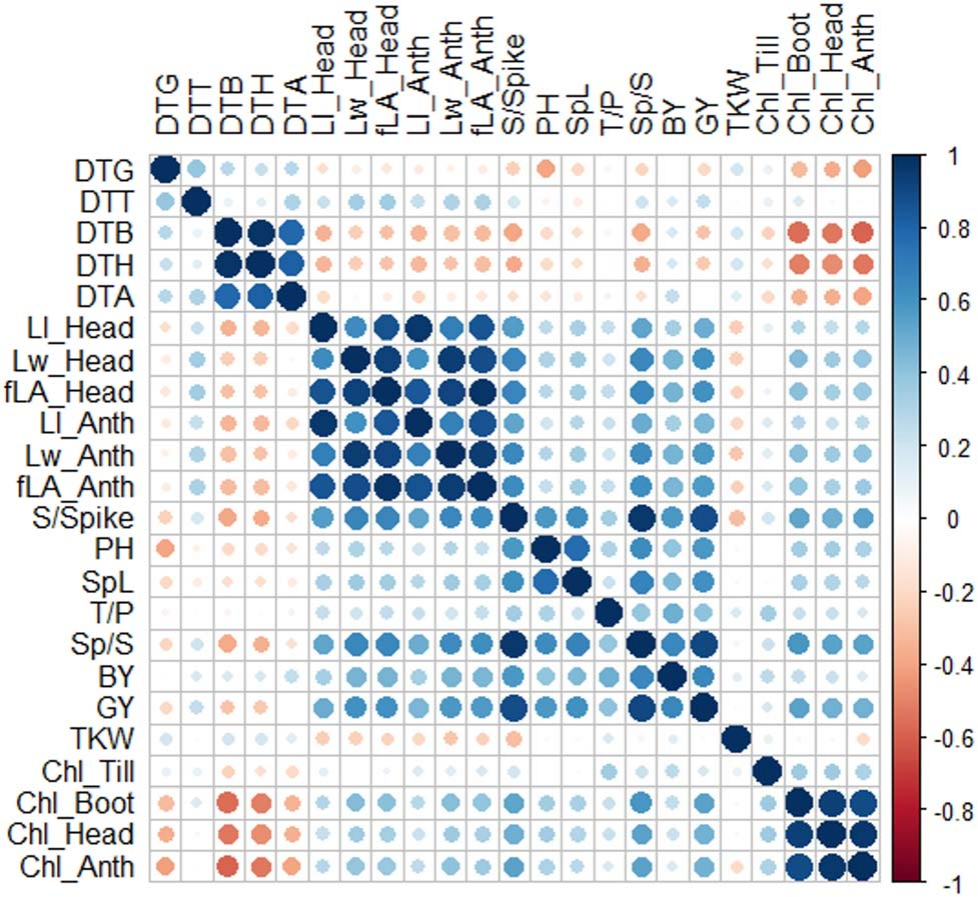
Correlation matrix of morpho-agro-physiological traits in wheat cultivars under nickel stress, visually representing Pearson correlation coefficients. Blue circles indicate positive correlations, and red circles indicate negative correlations, with the size and color intensity proportional to the strength of the correlation. This matrix highlights significant interdependencies among growth parameters, leaf morphology, spike attributes, yield components, and chlorophyll content, illustrating the influence of nickel stress on trait interactions.

The correlation analysis reveals significant interdependencies among morpho-agro-physiological traits under stress conditions (Fig. 3). DTG shows positive correlations with DTT (0.381**), DTB (0.278*), and DTA (0.282*), while correlating negatively with chlorophyll content at Chl_Boot (−0.319*), Chl_Head (−0.369**), and Chl_Anth (−0.413**). DTT is positively associated with Lw_Head (0.349**) and fLA_Head (0.345**). Strong correlations are seen between DTB and DTH (0.972**) and both with DTA (0.790** for DTB, 0.813** for DTH), with negative associations to Chl_Boot (−0.554**), Chl_Head (−0.524**), and Chl_Anth (−0.586** for DTB). Leaf morphology traits such as Ll_Head, Lw_Head, and fLA_Head correlate strongly with each other and with anthocyanin traits (*e.g.*, Lw_Anth 0.948**, fLA_Anth 0.973**), suggesting coordinated growth. S/Spike is highly correlated with yield traits BY (0.574**), GY (0.883**), and Sp/S (0.961**). PH has significant correlations with SpL (0.763**), BY (0.393**), and GY (0.572**). Chlorophyll traits across stages correlate closely, especially between Chl_Boot and Chl_Head (0.939**) and Chl_Anth (0.897**), indicating enhanced chlorophyll content supports growth and yield under stress (Table S1).

### 3.2. Soil analysis results

The soil sample exhibited notable chemical properties, with nitrates measured at 177.57 mg kg^−1^, indicating nutrient richness. The soil pH was moderately alkaline at 8.62, which influences specific nutrient availability and microbial community dynamics. Under such alkaline conditions, the solubility of essential micronutrients, particularly iron (Fe), zinc (Zn), copper (Cu), and manganese (Mn), is significantly restricted due to their precipitation into insoluble hydroxides or carbonates, while phosphorus often becomes immobilized by forming insoluble calcium phosphates.^51^ Furthermore, this elevated pH alters microbial activity by typically suppressing fungal diversity and favoring specific alkalitolerant bacterial communities, which directly impacts the rates of organic matter decomposition and soil enzyme functionality.^52^ EC was recorded at 0.435 mS cm^−1^, suggesting low salinity levels suitable for plant growth. Oxidizable carbon was at 0.98%, reflecting the organic matter decomposition rate. TOC and OM percentages were consistent at 2.26%, indicating a healthy organic content that supports soil structure and nutrient retention. Phosphates were measured at 11.33 mg kg^−1^, contributing to soil fertility, and are essential for plant growth. These parameters collectively indicate a nutrient-rich soil environment conducive to sustaining agricultural productivity.

### 3.3. Analysis of Ni uptake, accumulation, and translocation to aboveground parts of wheat

**Fig. 4:**
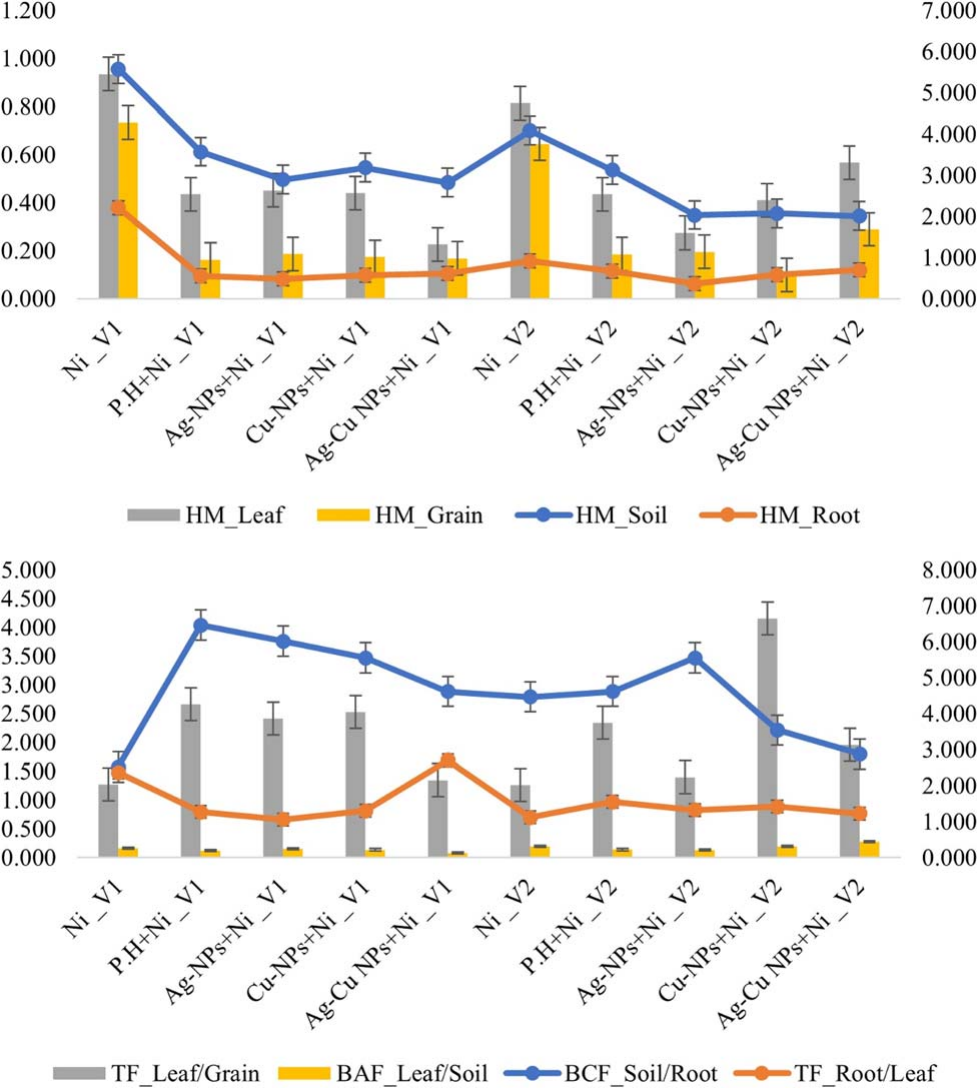
HM content and translocation factors across treatments in wheat cultivars V1 and V2 under various nanoparticle and Ni stress conditions. The left panel shows HM concentrations across matrices (HM-soil, HM-root, HM-leaf, and HM-grain) for both cultivars. The right panel presents BCF-soil/root, TF-root/leaf, TF-leaf/grain, and BAF-leaf/soil. Variations in HM uptake and translocation highlight the influence of nanoparticle treatments, with Cu-NPs and Ag–Cu NPs modulating Ni mobilization and accumulation in aerial parts compared to other treatments. Bars represent mean values ± SE. Abbreviations: heavy metal (HM), nickel (Ni), bioconcentration factor (BCF), translocation factor (TF), bioaccumulation factor (BAF), cultivar 1 (V1), cultivar 2 (V2), standard error (SE).

The analysis of Ni uptake, accumulation, and translocation based on the new data highlights distinct patterns in different plant parts across treatments, underscoring the differential responses of wheat cultivars V1 and V2. For cultivar V1, the highest Ni concentration in roots was observed in the Ni treatment alone (2.213 mg kg^−1^), while in leaves, Ni levels peaked in the Ni treatment (0.935 mg kg^−1^). Grain Ni accumulation in V1 was also highest under the Ni treatment (0.734 mg kg^−1^), with lower values recorded across other treatments, suggesting that Ni treatment alone drives higher root and grain accumulation. For cultivar V2, Ni concentration in roots reached its maximum (0.915 mg kg^−1^) under the Ni treatment, while Ag–Cu NPs + Ni resulted in the highest Ni levels in leaves (0.568 mg kg^−1^) and grains (0.290 mg kg^−1^). This pattern in V2 suggests that Ag–Cu NPs enhance Ni mobility to aerial parts compared to other treatments (Fig. 4).

The translocation and bioconcentration factors further illustrate the Ni movement. In V1, the TF-Root/Leaf was highest in Ag–Cu NPs + Ni (2.705), whereas TF-leaf/grain was most significant under P.H + Ni (2.666), showing treatment-specific translocation dynamics (Fig. 4). For V2, TF-root/leaf peaked in Cu-NPs + Ni (1.424), while TF-leaf/grain reached its maximum under the same treatment (4.155), indicating strong Ni movement from leaves to grains under Cu-NPs + Ni. The BAF varied across treatments, with V2 showing the highest BAF in Ag–Cu NPs + Ni (0.282) for leaf-to-soil accumulation, indicating its greater potential for Ni accumulation in aerial parts. In contrast, V1 had a maximum BAF of 0.168 in the Ni treatment alone, further highlighting cultivar- and treatment-specific accumulation and translocation tendencies. These findings reveal that nanoparticle treatments, especially Ag–Cu NPs and Cu-NPs, modulate Ni uptake, distribution, and bioaccumulation across wheat cultivars, with potential implications for managing Ni stress in plants. These findings reveal that nanoparticle treatments, especially Ag–Cu NPs and Cu-NPs, modulate Ni uptake, distribution, and bioaccumulation across wheat cultivars. While the increased translocation to leaves and grains suggests a degree of physiological tolerance, allowing the plant to redistribute ions and maintain metabolic functions under high stress, it also points to a critical trade-off. The enhanced Ni movement to edible grains, particularly in V2 under Ag–Cu NP treatment, signifies a potentially harmful metal redistribution that poses risks to food safety and human health. Therefore, while these nanoparticles effectively mitigate the growth-inhibiting effects of Ni, their role in facilitating metal transport to reproductive organs requires careful evaluation in the context of agricultural safety standards.

### 3.4. Metabolic studies

#### 3.4.1. Phytochemical content analysis of leaf

**Fig. 5:**
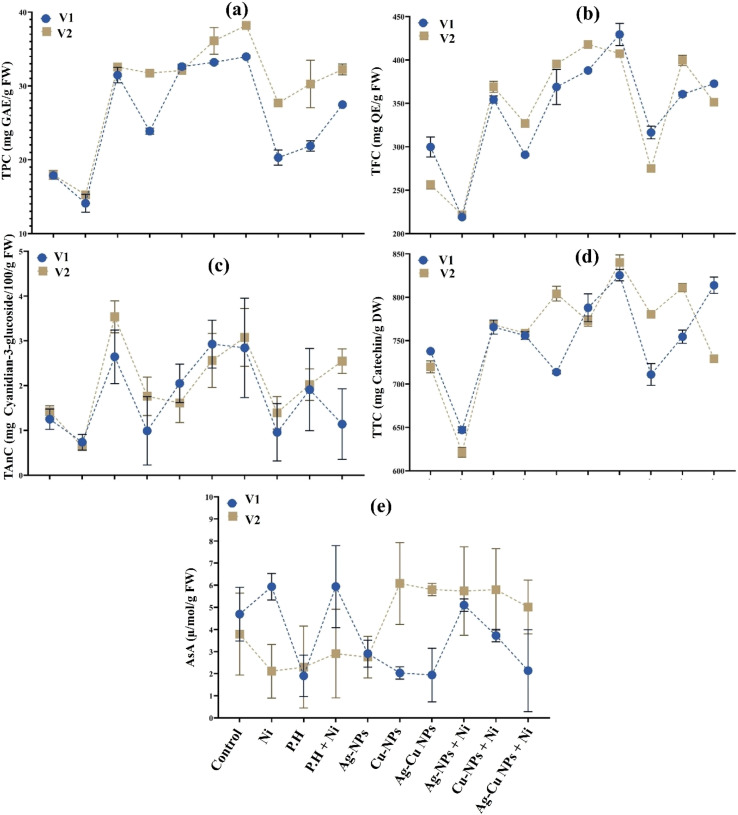
Phytochemical content in leaves of wheat varieties (V1 and V2) under various treatments. Mean ± SD plots with connecting lines show: (a) TPC, (b) TFC, (c) TAnC, (d) TTC, and (e) AsA across various treatments. The plots compare two experimental groups (V1 in blue and V2 in brown), illustrating the effects of treatments, including P.H, Ag-NPs, Cu-NPs, Ag–Cu NPs alone, and their combination with Ni, on different phytochemical parameters. Abbreviations: total phenolic content (TPC), total flavonoid content (TFC), total anthocyanin content (TAnC), total tannin content (TTC), ascorbic acid (AsA), gallic acid equivalent (GAE), quercetin equivalent (QE), fresh weight (FW), dry weight (DW).

Phytochemical content in leaf of wheat varieties (V1 and V2) under various treatments were shown in Fig. 5. In response to treatments, TPC exhibited significant variations, indicating enhanced phenolic compound accumulation under nanoparticle applications (Fig. 5a). Cu-NPs treatment led to the highest increase in TPC for both varieties, showing an 85.90% rise in V1 and an exceptional 101.11% increase in V2. Ag–Cu NPs also stimulated TPC accumulation, with 90.16% and 112.82% improvements in V1 and V2, respectively, suggesting their effective role in antioxidant defense under nickel stress. TFC followed this trend, with Cu-NPs again yielding maximum increments (29.38% in V1 and 63.08% in V2), emphasizing its role in bolstering flavonoid-based antioxidative responses as shown in Fig. 5b. The TAnC, which contributes to stress tolerance through ROS scavenging, increased substantially under Cu-NPs (134.82% for V1) and Ag–Cu NPs (116.86% for V2) as shown in Fig. 5c. TTC, another secondary metabolite associated with stress response, displayed moderate elevations, peaking at 16.72% under Ag–Cu NPs in V2 as depicts in Fig. 5d. AsA, a vital non-enzymatic antioxidant, saw the highest increase under Ag–Cu NPs for V2 (53.02%) as shown in Fig. 5e, highlighting its protective role against oxidative damage by directly scavenging ROS.

### 3.5. Chlorophyll and carotenoid content analysis

**Fig. 6:**
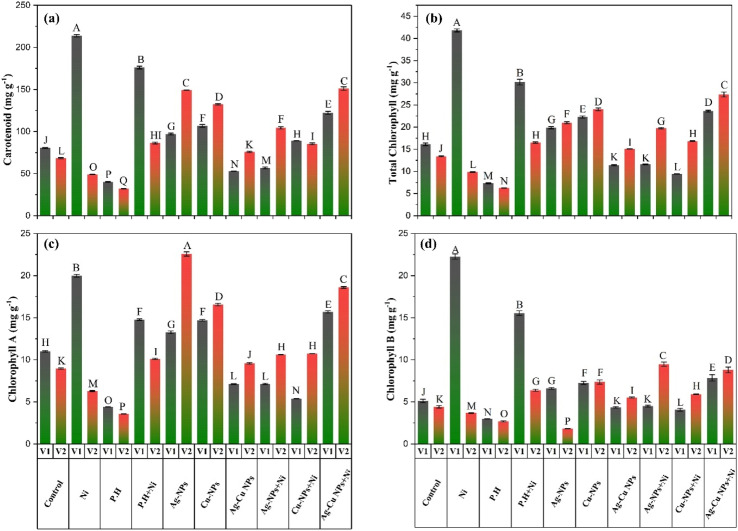
Carotenoid, total chlorophyll, chlorophyll a, and chlorophyll b content in wheat varieties (V1 and V2) under different treatments; (a) carotenoids, (b) total chlorophyll, (c) chlorophyll a, and (d) chlorophyll b Ni stress elevated carotenoids and chlorophyll in V1, while Ag–Cu NPs + Ni treatment boosted these pigments in V2. Ag and Cu-based NPs, especially Ag–Cu NPs + Ni, enhanced photosynthetic pigments in both varieties, indicating improved stress tolerance. Bars show mean values ± SE.

The analysis of chlorophyll and carotenoid content across different treatments revealed significant variations between the two wheat varieties, highlighting distinct physiological adaptation and mitigation mechanisms (Fig. 6). As shown in Fig. 6a, carotenoid levels peaked under Ni stress alone in V1 (∼214 mg g^−1^) and under the bimetallic Ag–Cu NPs + Ni treatment in V2 (∼152 mg g^−1^). The elevation of carotenoids in V1 under standalone metal stress points to an innate photoprotective mechanism, where carotenoids are upregulated to act as non-enzymatic antioxidants that scavenge singlet O_2_ and dissipate excess energy, thereby shielding the photosynthetic apparatus from nickel-induced photo-oxidation. A similar trend was observed for chlorophyll content. Chlorophyll a (Fig. 6c) was notably elevated under Ag-NPs treatment in V2 (∼22.76 mg g^−1^), while Cu-NPs + Ni treatment enhanced chlorophyll a across both cultivars. Chlorophyll b (Fig. 6d) and total chlorophyll (Fig. 6b) followed a parallel trajectory, reaching their maximum values in V1 under standalone Ni stress and in V2 under the synergistic Ag–Cu NPs + Ni application. Mechanistically, the robust recovery and enhancement of photosynthetic pigments mediated by the nanoparticles, particularly the bimetallic Ag–Cu framework, can be attributed to a dual-action defense system. Cu ions serve as an essential structural cofactor for plastocyanin in the thylakoid lumen and upregulate key antioxidant enzymes, effectively lowering structural oxidative stress. Simultaneously, Ag ions are known to inhibit ethylene biosynthesis and perception, thereby suppressing the activity of chlorophyll-degrading enzymes (chlorophyllase) and delaying leaf senescence under toxic metal stress. Together, this bimetallic synergy protects the structural integrity of the thylakoid membranes, restores electron transport efficiency, and upregulates pigment biosynthesis genes, ultimately driving higher stress tolerance in both wheat varieties.

### 3.6. Antioxidant capacity and activity

**Fig. 7:**
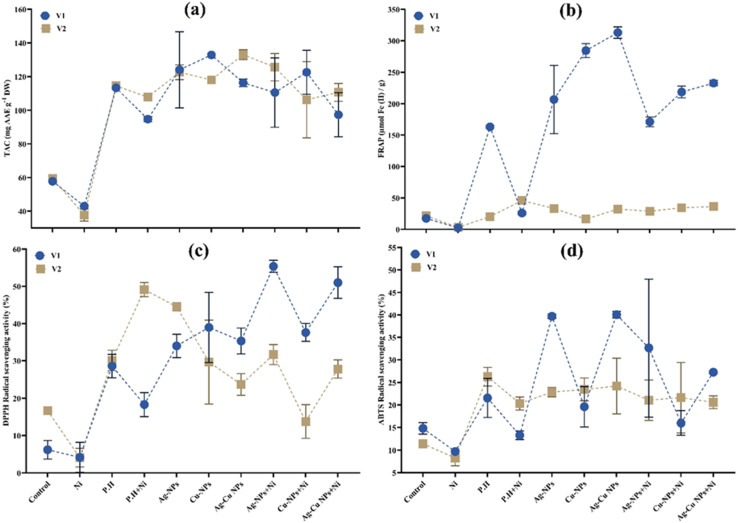
Antioxidant potential in wheat cultivars (V1 and V2) under various treatments. Mean ± SD plots with connecting lines represent: (a) TAC, (b) FRAP, (c) DPPH radical scavenging activity, and (d) ABTS radical scavenging activity. The plots compare two experimental groups (V1 in blue and V2 in brown), highlighting the effects of treatments, including P.H, Ag-NPs, Cu-NPs, Ag–Cu NPs alone, and their combination with Ni, on different antioxidant parameters. Abbreviations: total antioxidant capacity (TAC), ferric reducing antioxidant power (FRAP), 2,2-diphenyl-1-picrylhydrazyl (DPPH), 2,2′-azino-bis(3-ethylbenzothiazoline-6-sulfonic acid) (ABTS), ascorbic acid equivalent (AAE).

The antioxidant capacity and activity across different treatments showed significant variations in both wheat varieties (Fig. 7). TAC increased across nanoparticle treatments, underscoring an enhanced overall antioxidant defense. Cu-NPs treatment showed notable TAC improvements, with values rising by 129.90% in V1 and 98.18% in V2 (Fig. 7a), indicating that Cu-NPs significantly bolster the antioxidant capacity under stress. The FRAP, which assesses reducing potential, exhibited maximum improvement with Ag–Cu NPs in V1 (1699.80%) and Cu-NPs in V2 (53.93%) as depicts in Fig. 7b, suggesting increased electron-donating ability to counteract oxidative stress. In the DPPH assay, which measures free radical scavenging, Cu-NPs treatment stood out with a remarkable 527.57% increase in V1 (Fig. 7c). Similarly, the ABTS assay demonstrated maximum effectiveness in Ag–Cu NPs treatments, achieving 170.40% in V1 as shown in Fig. 7d, indicative of strong radical neutralization capabilities, which are critical in minimizing oxidative stress.

#### 3.6.1. Oxidative stress indicators

**Fig. 8:**
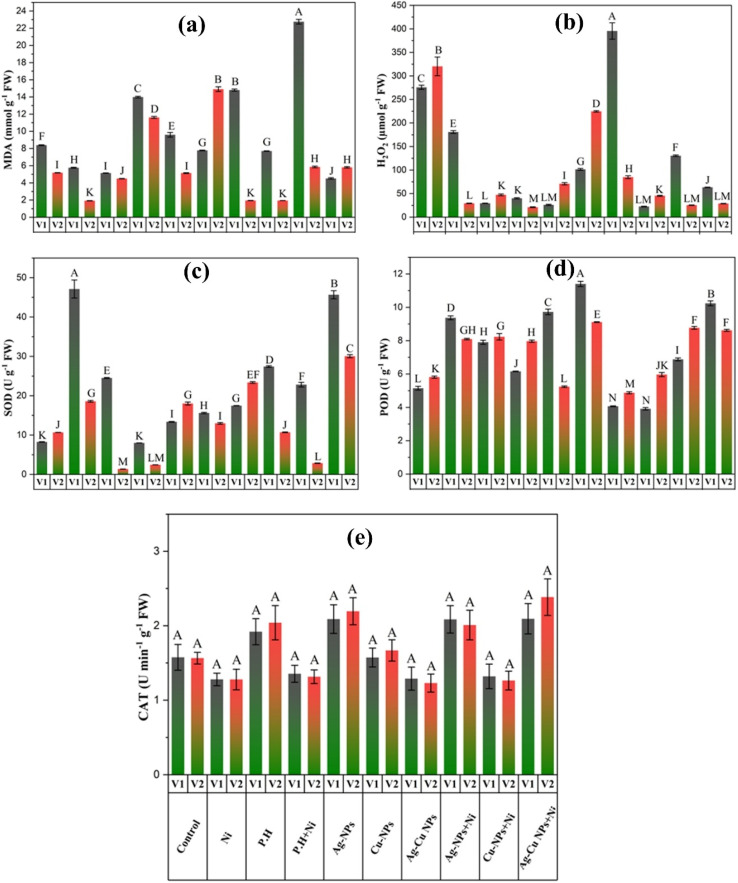
Oxidative stress markers and enzymatic antioxidant activities in wheat cultivars under various treatments. Panels show levels of (a) MDA, (b) H_2_O_2_, (c) SOD, (d) POD, and (e) CAT in cultivars V1 and V2. Ni stress significantly elevated MDA and H_2_O_2_ levels, indicating severe oxidative damage, particularly in V1. Antioxidant enzymes increased notably under Ag- and Cu-based nanoparticle treatments, with Ag–Cu NPs + Ni showing the most effective mitigation of oxidative stress across both cultivars. Bars represent mean values ± SE. Abbreviations: malondialdehyde (MDA), hydrogen peroxide (H_2_O_2_), superoxide dismutase (SOD), peroxidase (POD), catalase (CAT), variety 1 (V1/SKD-1), variety 2 (V2/Borlaug-16).

MDA levels, an indicator of lipid peroxidation, were reduced in several treatments, especially under Ag-NPs and Cu-NPs in V1, with reductions of −38.49% and −46.18%, respectively, as shown in Fig. 8a. This significant decrease in MDA levels directly implies lower oxidative damage to cell membranes. Biochemically, this protection is explicitly justified by the concomitant upregulation of the enzymatic antioxidant system (Section 3.6.2); the elevated activities of SOD (+90.72%) and POD (+122.05%) effectively intercept and scavenge ROS, such as superoxide radicals (O_2_˙^–^) and H_2_O_2_, before they can initiate the radical chain reactions of lipid peroxidation on polyunsaturated fatty acids within the cellular membranes.^53^ This mechanistic correlation is further supported by our correlation analysis (Fig. 2), which demonstrates a robust negative relationship between active antioxidant defense traits and lipid peroxidation markers. Hydrogen peroxide (H_2_O_2_) levels, which signify acute oxidative stress, dropped markedly in the Cu-NPs treatment for V1 (−82.71%) as depicted in Fig. 8b, highlighting the treatment's efficiency in managing oxidative stress by reducing H_2_O_2_ accumulation.^54,55^ The strong negative correlation between the high percentage increases in enzyme activities and the sharp decline in MDA and H_2_O_2_ levels confirms that the induced enzymatic activities reached a functional effectiveness level capable of maintaining membrane integrity and cellular homeostasis under Ni stress.

#### 3.6.2. Enzymatic antioxidants

The activities of key enzymatic antioxidants, including POD, SOD, and CAT, were measured to assess enzymatic defenses against oxidative damage. SOD, essential for converting superoxide radicals to less harmful forms, displayed a similar pattern, with a 90.72% increase in V1 under Cu-NPs as depicts in Fig. 8c. POD activity was significantly enhanced under Cu-NPs for V1, reaching an increase of 122.05% as shown in Fig. 8d, which suggests a robust response in breaking down peroxides generated by stress. The biological relevance of these substantial increases in enzymatic activity lies in their ability to cross a functional threshold necessary for systemic detoxification. By significantly upregulating SOD and POD, the plants treated with Cu-NPs effectively bridge the gap between ROS generation and scavenging, providing a metabolic buffer that prevents irreversible cellular damage. CAT activity, however, was generally reduced across treatments (Fig. 8e), which may reflect altered H_2_O_2_ decomposition dynamics under nickel stress. Nonetheless, Cu-NPs managed to maintain moderate CAT activity in V1, supporting the plant's ability to detoxify H_2_O_2_ despite overall stress impacts on CAT.

### 3.7. Correlation analysis of antioxidant enzymes, pigments, and oxidative stress markers under nickel stress

**Fig. 9:**
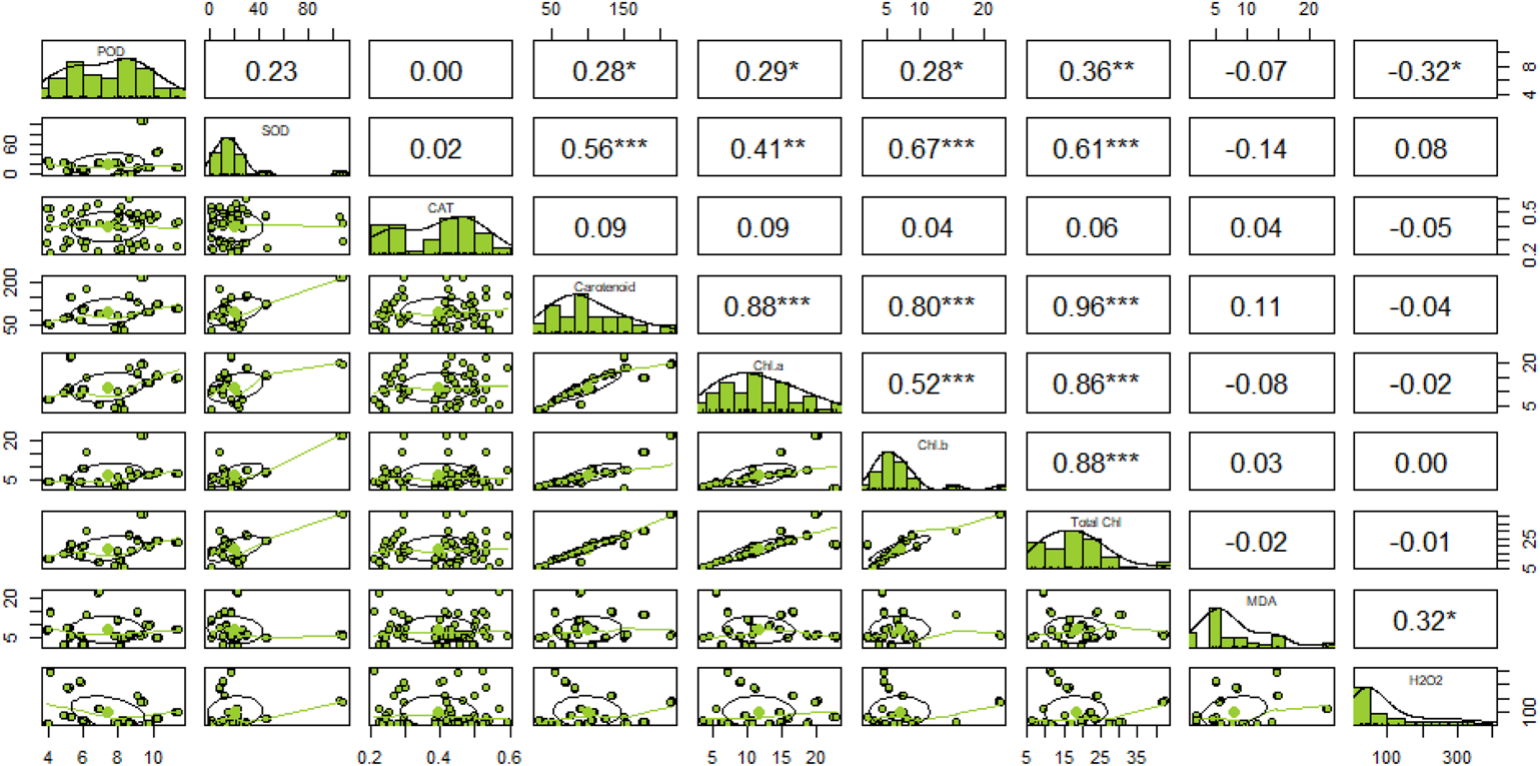
Scatterplot matrix displaying pairwise correlations among antioxidant enzymes, photosynthetic pigments, and oxidative stress markers in wheat cultivars under nickel stress. The matrix includes histograms along the diagonal, representing the distribution of each variable, and scatterplots with fitted lines illustrating relationships between pairs of variables. Positive and negative correlation coefficients are displayed in each cell, with significant correlations marked at *p* < 0.05 (*), *p* < 0.01 (**), and *p* < 0.001 (***), highlighting key interactions involved in stress tolerance mechanisms.

The correlation analysis among physiological and biochemical parameters under stress reveals several significant relationships (Fig. 9). POD shows positive correlations with carotenoids (0.278*), Chl.a (0.292*), Chl.b (0.278*), and total Chl (0.359**), while negatively correlated with H_2_O_2_ (−0.324*). SOD is positively correlated with carotenoids (0.558**), Chl.a (0.408**), Chl.b (0.669**), and Total Chl (0.612**). Carotenoids exhibit strong positive correlations with Chl.a (0.884**), Chl.b (0.801**), and total Chl (0.956**), highlighting interconnected pigment synthesis. Chl.a and Chl.b are also positively associated (0.519**) and correlate with total Chl (0.858** and 0.880**, respectively). MDA shows a positive correlation with H_2_O_2_ (0.320*), indicating oxidative stress markers' relationship. These findings highlight the interplay among antioxidant enzymes, pigments, and stress markers under stress conditions (Table S2).

### 3.8. Grain nutrition assessment and hierarchical heatmap analysis

**Fig. 10:**
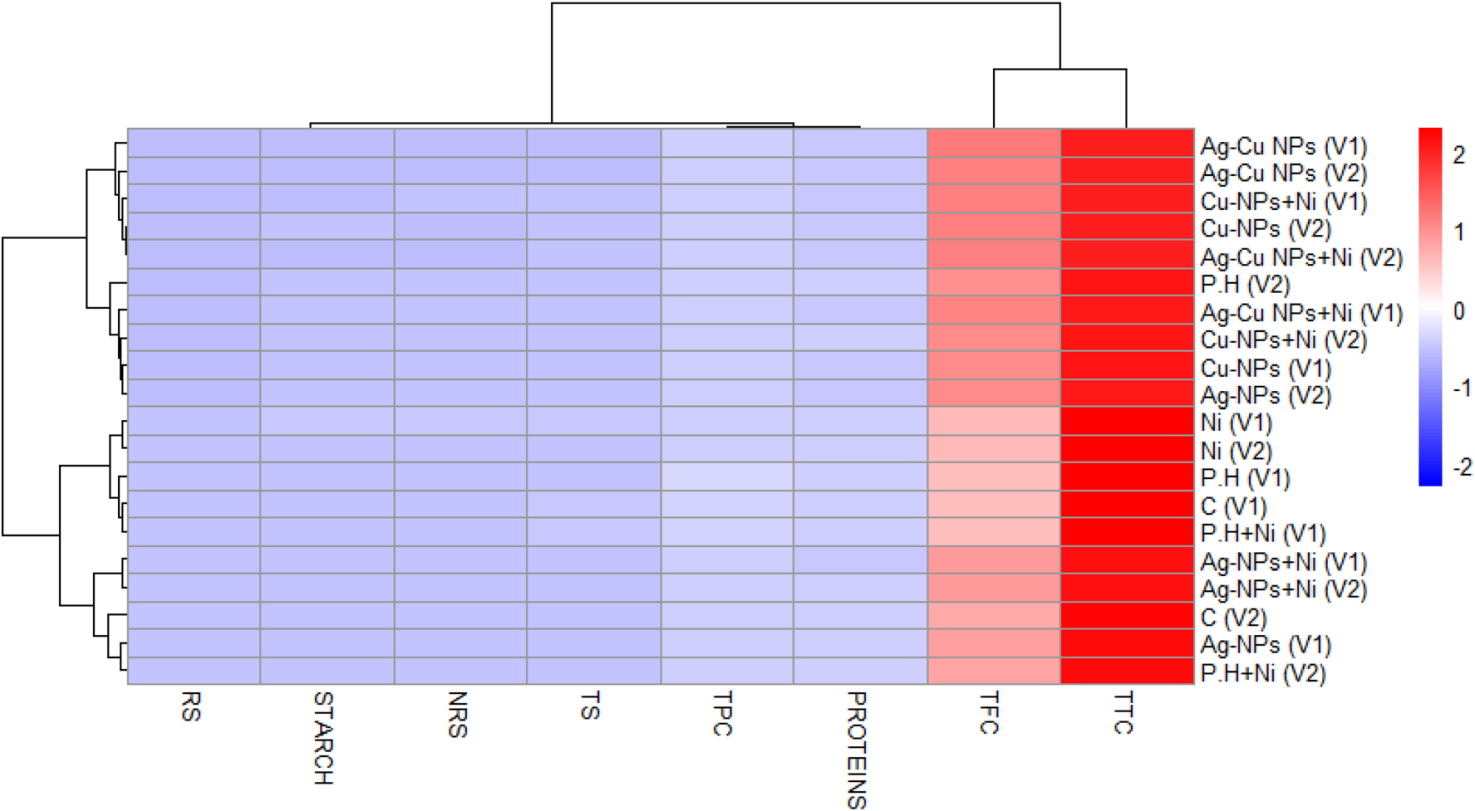
Heatmap showing the accumulation of biochemical compounds in wheat grains of cultivars V1 and V2 under different nanoparticle and nickel treatments. Columns represent grain compounds and rows display treatment responses, with blue indicating lower and red indicating higher concentrations of each compound. Abbreviations: reducing sugars (RS), non-reducing sugars (NRS), total sugars (TS), total phenolic content (TPC), total flavonoid content (TFC), total tannin content (TTC).

To comprehensively evaluate the nutritional shifts and metabolic adaptations in wheat grains under various treatments, a hierarchical clustering heatmap was constructed (Fig. 10), mapping the accumulation patterns of key primary and secondary biochemical compounds across both cultivars (V1 and V2).

#### 3.8.1. Grain phytochemical content and secondary metabolites

Significant differences were observed in grain phytochemical content across treatments. TPC increased notably under Ag–Cu NPs treatment for both varieties, with an increase of 51.83% in V1 and 62.66% in V2 (Fig. S1). This suggests strong antioxidative potential induced by Ag–Cu NPs. TFC also peaked under Ag–Cu NPs, with increases of 94.44% in V1 and 63.19% in V2, highlighting the nanoparticles' effectiveness in enhancing flavonoid synthesis. TTC, another indicator of antioxidant properties, showed maximum increases under Ag–Cu NPs for both varieties, with 12.11% for V1 and 15.69% for V2, indicating its contribution to stress tolerance. Significant differences were observed in grain phytochemical profiles across treatments, with the heatmap revealing a prominent cluster of highly elevated secondary metabolites across almost all experimental groups (Fig. 10 and S1). TPC increased notably under standalone Ag–Cu NPs treatment for both varieties, showing an increase of 51.83% in V1 and 62.66% in V2. Similarly, TFC peaked sharply under Ag–Cu NPs, with substantial increases of 94.44% in V1 and 63.19% in V2. TTC, another essential indicator of secondary antioxidant properties, reached its maximum accumulation under the bimetallic Ag–Cu NPs treatment, showing a 12.11% increase in V1 and a 15.69% increase in V2. As illustrated by the column dendrogram in Fig. 10, TFC and TTC group together into a separate, distinct metabolic block characterized by intense red coloration across almost all rows. This strong global upregulation indicates that the biosynthesis of secondary phenylpropanoid defense paths is universally triggered in the grain under nano-elicitation and stress conditions to form a robust non-enzymatic antioxidant barrier against heavy metal toxicity.

#### 3.8.2. Carbohydrate profile and primary energy storage

In contrast to the secondary defense metabolites, primary nutritional components, including RS, starch, NRS, and TS, clustered tightly together on the left side of the heatmap, showing a predominantly suppressed accumulation profile represented in shades of blue (Fig. 10). Carbohydrate composition varied across treatments, reflecting differences in energy allocation and metabolic partitioning under stress. In V1, Ag–Cu NPs treatment minimized carbohydrate loss, with NRS showing a minimal reduction of only −0.93%, demonstrating superior carbohydrate retention compared to other groups. Conversely, the Cu-NPs + Ni treatment in V2 led to much more substantial reductions, with NRS and RS falling by −20.37% and −14.25%, respectively (Fig. 10). TS followed a parallel trajectory; grains treated with Ag–Cu NPs under stress in V2 exhibited the smallest overall decrease (−1.54%), highlighting the unique potential of this bimetallic formulation to preserve primary carbon reserves. Starch levels remained remarkably stable under Ag–Cu NPs in V1 (+0.73%), whereas other metal stress treatments caused severe depletion, suggesting that the bimetallic framework helps stabilize the grain's energy reserves by maintaining starch biosynthetic enzyme activities under toxicity.

#### 3.8.3. Protein content and treatment clustering

Protein content, a critical determinant of grain nutritional quality, showed distinct treatment-dependent modulation. Standalone Ag–Cu NPs increased grain protein content by 7.00% in V1 and stabilized it in V2 (−0.19%). In stark contrast, standalone nickel stress (Ni) caused severe damage to protein synthesis, slashing levels by −26.96% in V1 and −15.39% in V2. This dramatic drop is clearly visible in the rows corresponding to standalone Ni stress, which exhibits a strong blue color shift across all primary metabolites and protein coordinates (Fig. 10). The row dendrogram in Fig. 10 provides key insights into how these treatments group organically. Standalone Ag–Cu NPs and Cu-NPs treatments cluster near the top, characterized by optimal protein and carbohydrate maintenance. Meanwhile, the standalone Ni stress treatments group closely with the P.H + Ni, Ag-NPs + Ni, and Cu-NPs + Ni co-treatments. This clustering pattern demonstrates that while individual nanoparticle applications (especially Cu and Ag–Cu) provide clear metabolic protection, the structural presence of Ni remains a dominant stress driver that forces the plant to prioritize secondary defense allocation (TFC and TTC) at the expense of primary grain storage (proteins and carbohydrates).

### 3.9. Principle component analysis

**Fig. 11:**
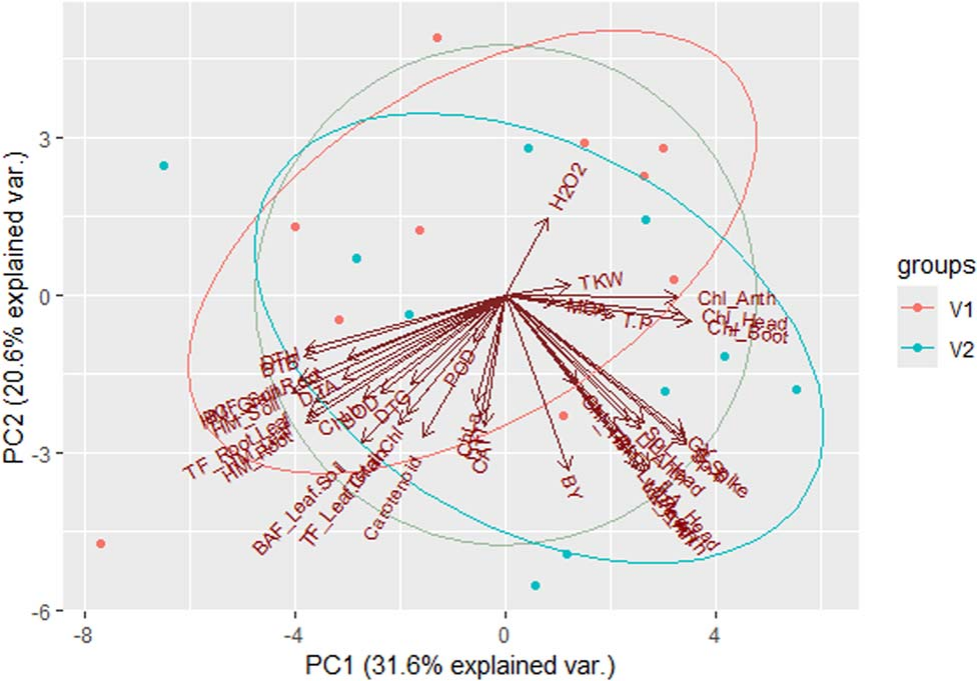
PCA biplot illustrating trait interactions among wheat cultivars V1 and V2 under nickel and nanoparticle treatments. Trait vectors display directional influence, with chlorophyll parameters aligning with V1 (SKD-1) and oxidative stress markers and translocation factors aligning with V2 (Borlaug-16). The 95% confidence ellipses indicate distinct clustering between cultivars.

The PCA analysis reveals distinct interactions among morpho-agro-physiological and biochemical traits under nickel and nanoparticle treatments in wheat cultivars V1 and V2. Positive interactions are observed between chlorophyll content (Chl-Boot, Chl-Head, Chl-Anth) and cultivar V1, indicating enhanced photosynthetic traits in response to treatments. Oxidative stress markers such as H_2_O_2_ and TF-root/leaf, TF-leaf/grain show a strong positive alignment with cultivar V2, suggesting that V2 is more responsive to treatments affecting metal translocation and oxidative stress management. Negative interactions are implied between photosynthetic parameters and oxidative stress markers, as they are oriented in opposite directions, highlighting that increased stress responses (like elevated H_2_O_2_) are associated with reduced chlorophyll levels (Fig. 11). TKW exhibits moderate positive interactions with V2, indicating a slight enhancement in yield potential under stress conditions for this cultivar. Meanwhile, certain traits such as CAT and POD do not show a strong association with either cultivar, indicating minimal or no interaction with the treatments in influencing cultivar-specific responses. Overall, this analysis underscores that V1's response is characterized by enhanced chlorophyll content, while V2 displays stronger metal translocation and stress resilience traits.

### 3.10. Comparative analysis of cultivar-specific tolerance

The experimental data and PCA results reveal a clear divergence in how the two wheat cultivars utilize nanoparticles to manage nickel stress. V1 primarily exhibits a “biochemical defense” strategy, characterized by its superior enzymatic upregulation, where SOD and POD activities increased by 90.72% and 122.05% respectively, leading to the most significant reduction in H_2_O_2_ accumulation (−82.71%). In contrast, V2 demonstrates a “yield-priority” strategy, maintaining a higher absolute grain yield of 0.91 g and a 21.3% improvement in yield stability, despite having a higher leaf-to-grain translocation factor for Ni (4.155). These results indicate that while SKD-1 focuses on cellular detoxification and membrane protection, Borlaug-16 prioritizes reproductive output and biomass partitioning, suggesting that nanoparticle effectiveness is influenced by the specific genetic resilience of the cultivar.

## 4. Discussion

Nickel stress is widely recognized for its detrimental impact on plant growth and agronomic traits, particularly in wheat, where it disrupts photosynthesis, reduces biomass, and ultimately hampers yield.^56^ In this study, both wheat cultivars experienced significant declines in plant height, leaf area, and grain yield under Ni stress, especially in untreated conditions. These results align with findings by ref. 3 and 57, who observed similar growth suppression under heavy metal stress. This growth inhibition is directly driven by Ni-induced oxidative stress, cellular dysfunction, and severe nutrient uptake disruption;^58,59^ excessive cellular Ni substitutes essential divalent cations (such as Mg^2+^ and Fe^2+^) in vital metalloenzymes, inducing systemic metabolic imbalances. These physiological disruptions directly interfere with chlorophyll synthesis and hinder the efficiency of photosynthesis, cascading into stunted plant development and restricted grain yield. Understanding abiotic stress responses in higher plants is important to develop measures for mitigating its impact on crop yield. Nanoparticles are currently gaining attention due to their capacity in plant stress mitigation by increasing plant resilience and contributing to plant recovery.^60^ The multi-level effects of NPs on wheat resilience involve a complex interplay of cellular, physiological, and whole-plant mechanisms. At the cellular level, Cu and Ag–Cu NPs serve as nano-regulators that stabilize the plasma membrane and reduce lipid peroxidation, as evidenced by the marked decrease in MDA levels. Physiologically, these NPs enhance the efficiency of the photosynthetic apparatus by protecting the oxygen-evolving complex and improving the electron transport rate between photosystem II and I.^61,62^ Furthermore, the NPs trigger a systemic signaling response that upregulates the production of secondary metabolites, such as phenolics and flavonoids, which act as internal buffers against Ni-induced toxicity.^63^ At the whole-plant level, the NPs modify the source–sink relationship, ensuring that even under metal stress, nutrient mobilization towards the developing grains is maintained, thereby stabilizing the final yield. These diverse effects across various biological levels underscore the potential of nano-priming as a holistic strategy for crop protection in contaminated environments.^64,65^ Recent reviews highlight that nano-enabled agriculture offers a transformative approach to improving crop tolerance through the precise delivery of nutrients and the modulation of plant signaling pathways, which are essential for maintaining productivity under environmental constraints.^66–68^ This study shows that Cu-NPs and Ag–Cu bimetallic NPs, significantly improved morphological traits such as plant height, spike length and yield parameters in wheat cultivars under Ni stress. This is consistent with the findings of ref. 22 and 69, which demonstrate that NPs support growth and recovery from oxidative stress and nutrient acquisition.

Mechanistically, it remains a point of investigation whether NPs modify Ni behavior through direct chemical sequestration in the soil or through internal physiological transport. Our findings suggest that while some degree of soil-level immobilization may occur, the nanoparticles primarily influence Ni dynamics through altered uptake and physiological transport processes. The application of Cu and Ag–Cu bimetallic NPs appears to modulate the expression of metal transporter proteins and promote the internal redistribution of Ni ions, as evidenced by the significant changes in TF observed in both cultivars. The mechanisms responsible for those improvements involve antioxidant behavior of NPs contributing to ROS scavenging in conditions of Ni stress. Recent studies have found that NPs stabilize cellular structures, enhance nutrient mobilization to support cellular resilience and protect of photosynthetic processes.^70^ A critical and unconventional observation in this study is the vastly increased Ni translocation to leaves and grains induced explicitly by the bimetallic Ag–Cu NPs (Fig. 4). This directly contrasts with a large body of classical nano-remediation literature^71–73^ which typically posits that nanoparticles mitigate heavy metal toxicity by structurally trapping or sequestering toxic ions within root vacuoles, thereby minimizing dangerous translocation to upper aerial and reproductive tissues. However, our results closely mirror an emerging, alternative toxicological paradigm reported by ref. 74 and multi-metal models by ref. 75. These studies demonstrate that certain green-synthesized bimetallic configurations can act as high-efficiency ionic carriers or significantly alter xylem loading dynamics by upregulating oligopeptide transporters (OPT) and yellow stripe-like (YSL) transporter families. This increased translocation represents an advanced level of physiological tolerance, where the plant successfully maintains structural and photosynthetic integrity despite higher internal metal loads. However, it also introduces a critical food-safety conflict with conventional literature that warns against promoting heavy metal bioaccumulation inside the edible portions of food crops. Chlorophyll biosynthesis is significantly disrupted under heavy metal stress, especially in response to Ni exposure, and photosynthetic efficiency is impaired. As a result of Ni toxicity, chloroplast formation and function is impeded as the chlorophyll content is reduced and rates of photosynthesis are lowered, which in turn affects growth and yields.^76^ This improvement is attributed to the ability of nanoparticles to act as “artificial light-harvesting complexes” or by stimulating the expression of genes involved in the Calvin cycle and light-dependent reactions.^61^ Specifically, Cu-NPs serve as essential components of plastocyanin, an integral electron carrier in the photosynthetic electron transport chain,^77^ thereby directly enhancing the quantum yield of photosystem II (PSII). Furthermore, Ag-NPs have been reported to increase the number of chloroplasts per cell and expand the size of the grana, which provides a larger surface area for light absorption.^78,79^ By stabilizing the oxygen-evolving complex and protecting the D1 protein from oxidative degradation, these nanoparticles ensure that the photosynthetic machinery remains functional even under the pressure of Ni-induced ROS.^80^ Despite the clear agronomic benefits observed, the potential risks related to field-scale application must be addressed. The long-term accumulation of silver and copper nanoparticles in agricultural soils could lead to secondary toxicity, affecting soil microbial communities and soil health. Furthermore, the enhanced movement of Ni into wheat grains, as observed in our Ag–Cu bimetallic NPs treatments, poses a significant risk of food-chain transfer. These findings emphasize that while NPs are effective mitigation tools, their application must be carefully regulated to prevent the entry of toxic metals into the human diet through biomagnification. Such recovery is consistent with previous findings showing that NPs can support oxidative stress recovery and retention of chlorophyll through the upregulation of chlorophyllide oxygenase and other biosynthetic enzymes.^81,82^ It is likely that the photoprotective effects of NP in the leaves are either through stabilization of cellular membranes or up regulation of antioxidant enzyme activities to protect against chlorophyll degradation.^83^ Therefore, Cu and Ag–Cu NPs may represent advantageous agents to mitigate Ni stress in plants, assisting plants to keep chlorophyll levels and photosynthetic efficiency improving subsequently plant health and productivity under adverse conditions.^84,85^

Heavy metals such as Ni generate oxidative stress in plants and as a primary defense mechanism, plants produce antioxidants. When Ni stress environments are encountered, ROS are over produced and can lead to oxidative damage of cellular components, causing an upregulation of antioxidant compounds.^86^ Here, within this study, only Ni stress increased enzymatic antioxidant activity while inclusion of Cu and Ag–Cu bimetallic NPs further enhanced levels of phenolic compounds, flavonoids, and certain antioxidant enzymes. The notion that the enhance in antioxidant amounts beneath NP therapies is similar to outcomes in ref. 87, who reported a similar boost in phenolic and flavonoid content as well as enzymatic antioxidants in Ni-stressed plants treated with NPs. These antioxidants play a crucial role in neutralizing ROS, thereby mitigating oxidative stress and contributing to cellular stability.

It may be that the ROS scavenging capacity of Cu and Ag–Cu bimetallic NPs enables their use in enhancing plant antioxidant responses and prevent cellular damage and assist plant defense mechanisms. These nanoparticles have been shown to reduce oxidative stress *via* stabilizing cellular membranes and protecting biomolecules from degradation.^85^ Cu-NPs additionally enhance key enzyme activities such as POD and SOD, which are needed for ROS detoxification.^88^ NPs add to the defense of the adverse effects of Ni stress by reinforcing both nonenzymatic antioxidants (such as phenolics and flavonoids) and enzymatic antioxidants (POD, SOD). This dually performing role of NPs as used both for scavenging ROS and the reinforcement of antioxidant activity render.^89^ Key biomarkers for cell damage due to heavy metal stress are oxidative stress indicators, *e.g.*, MDA and H_2_O_2_. These indicators are elevated by Ni exposure, like their induction in response to oxidative stress in plant cells leading to increased lipid peroxidation. Application of Cu-NPs and Ag–Cu bimetallic NPs notably reduced both MDA and H_2_O_2_ levels, suggesting oxidative damage mitigation ability. These are similarly as expected findings related to ref. 90, demonstrating that nanoparticles are successful can diminish oxidative pressure markers and empower vegetation resilience to metal toxicity.

Nanoparticle treatments are demonstrated to reduce MDA and H_2_O_2_ due to their capacity to stabilize cellular membranes and augment the antioxidant defenses, minimizing lipid peroxidation and chloroplast structures. It seems that nanoparticles can trigger antioxidant enzyme pathway and helping in ROS scavenging to preserve the structural integrity of plants under stresses.^85^ Cu and Ag–Cu bimetallic NPs not only suppress oxidative damage but extend Cu and Ag transport to the photosystem resulting in improved nutrient uptake and photosynthetic performance under Ni stress, enhancing plant tolerance on adverse effects of Ni. The potential that nanoparticles can serve as useful tools in improving stress tolerance in plants, through biochemical stabilization and prevention of oxidative damage is highlighted by this.

## 5. Conclusion

This study demonstrates significant improvements in key agronomic traits, including grain yield, chlorophyll content, and oxidative stress reduction, in wheat under nickel stress. The application of *Peganum harmala*-synthesized Ag, Cu, and bimetallic Ag–Cu NPs effectively enhanced wheat tolerance to heavy metal toxicity by strengthening antioxidant defense, improving phytochemical accumulation, and stabilizing physiological functions. Among these, silver–copper nanoparticles showed the highest efficacy in mitigating oxidative damage, though they also facilitated the translocation of Ni to the grains. While these biochemical results provide strong evidence of stress mitigation, we recognize that a full mechanistic understanding requires the exploration of the genetic machinery. Therefore, this research serves as a foundation for future field-scale studies that will incorporate molecular-level work, including gene expression analysis of metal transporters and transcriptomic profiling, to confirm the genomic pathways involved in nanoparticle-mediated tolerance. The cultivar-specific adaptation strategies identified through this study offer valuable insights for breeding and precision agronomy. These findings highlight green nanotechnology as a sustainable and effective approach to enhancing crop resilience, provided that nanoparticle scalability and long-term environmental impacts are monitored to ensure food safety and environmental health.

## Author contributions

A. K.: conceptualization, data curation, investigation, methodology, software, and writing – original draft and funding acquisition. M. A.: data curation, formal analysis, software, visualization, and writing – review & editing. T. A. Q. and R. A. K.: data curation and formal analysis, and writing – review & editing. B. A.: data curation and formal analysis and resources. D. H. and A. S.: data curation, formal analysis, writing – review & editing. Z. K. S.: conceptualization, funding acquisition, data curation, project administration, resources, supervision, validation, and writing – review & editing. M. M.: project administration, formal analysis, resources, supervision. All authors reviewed the results and approved the final version of the manuscript.

## Conflicts of interest

The authors declare no competing interests.

## Supplementary Material

RA-016-D6RA03724K-s001

## Data Availability

The data that support the findings of this study are available within the article and its associated supplementary information (SI). Supplementary information: Pearson correlation coefficients among morpho-agro-physiological traits (Table S1), Pearson correlation coefficients among biochemical antioxidant enzymes and oxidative stress markers (Table S2), and a graphical analysis of the phytochemical and nutritional content in wheat seed varieties under various nanoparticle treatments (Fig. S1). See DOI: https://doi.org/10.1039/d6ra03724k.
