# Supplementary material for: Peganum harmala-mediated green synthesis of Ag, Cu, and Ag–Cu bimetallic nanoparticles for the chemical mitigation of nickel-induced oxidative stress in Triticum aestivum L
Source: RSC Adv. 2026 Jul 3;16(35):35919–36. doi: 10.1039/d6ra03724k (PMC13330172; doi:10.1039/d6ra03724k)
Supplement: RA-016-D6RA03724K-s001 [file RA-016-D6RA03724K-s001.pdf]

Supplementary Information (SI) for RSC Advances.
This journal is © The Royal Society of Chemistry 2026

Peganum harmala-mediated green synthesis of Ag, Cu, and Ag-Cu bimetallic nanoparticles for the chemical mitigation of nickel-induced oxidative stress in _Triticum aestivum_ L.

Amjid Khan[1,2*], Tauqeer Ahmed Qadri[1,2,3], Rashid Abbas Khan[1,2], Muhammad Anas[4], Dilawar Hassan[1,2], Ayesha Sani[1,2], Bushra Ashiq[1,2,5], Zabta Khan Shinwari[6,7*], Malik Maaza[1,2]

[1]UNESCO-UNISA Africa Chair in Nanosciences and Nanotechnologies, College of Graduate Studies, University of South Africa, 1 Preller Street, Muckleneuk Ridge, P.O. Box 392, Pretoria, Gauteng Province, 0003, South Africa

[2]African Centre of Competencies in Enhanced Nanosciences & Nanotechnologies for SDGs (ACCENTS), 1 Preller Street, Muckleneuk Ridge, P.O. Box 392, Pretoria, Gauteng Province, 0003, South Africa

[3]Department of Biosciences, COMSATS University Islamabad, Islamabad, 45550, Pakistan

[4]Department of Soil Science & Plant Nutrition, Selçuk University Campus, Konya, 42079, Türkiye

[5]Department of Biomedical Engineering, Research Center for Nano-biomaterials & AMP; Regenerative Medicine, College of Artificial Intelligence, Taiyuan University of Technology, Taiyuan, 030024, PR China

[6]Department of Plant Sciences, Faculty of Biological Sciences, Quaid-i-Azam University, Islamabad, 45320, Pakistan

[7]Federal Urdu University of Arts, Sciences and Technology (FUUAST), Karachi, 75300, Pakistan

***Corresponding Author:** Amjid Khan. Email: khana2@unisa.ac.za; Zabta Khan Shinwari. Email: shinwari@qau.edu.pk

Table S1: Pearson correlation coefficients among morpho-agro-physiological traits in wheat cultivars under nickel stress, highlighting significant relationships among growth parameters, leaf morphology, spike traits, yield components, and chlorophyll content at various stages. *Significant correlations are denoted at the 0.01 (**) and 0.05 (*) levels, indicating interdependencies that contribute to growth and stress resilience.*

Parameters	DTG	DTT	DTB	DTH	DTA	Ll_Head	Lw_Head	fLA_Head	Ll_Anth	Lw_Anth	fLA_Anth	S/Spike	PH	SpL	T/P	Sp/S	BY	GY	TKW	Chl_Till	Chl_Boot	Chl_Head	Chl_Anth
**DTG**	1	.381**	.278*	0.228	.282*	-0.173	-0.100	-0.128	-0.119	-0.081	-0.095	-0.238	-.397**	-0.206	-0.051	-0.218	0.013	-0.203	0.181	0.092	-.319*	-.369**	-.413**
**DTT**	.381**	1	0.089	0.124	.302*	0.221	.349**	.345**	0.238	.302*	.313*	0.177	-0.075	-0.093	-0.011	0.204	0.168	0.243	0.027	0.091	0.153	0.046	0.015
**DTB**	.278*	0.089	1	.972**	.790**	-.341**	-0.248	-.294*	-.337**	-.299*	-.316*	-.384**	-0.191	-0.173	-0.058	-.374**	0.160	-.289*	0.190	-0.223	-.554**	-.524**	-.586**
**DTH**	0.228	0.124	.972**	1	.813**	-.332**	-0.247	-.286*	-.325*	-.289*	-.303*	-.371**	-0.182	-0.173	-0.026	-.354**	0.171	-.263*	0.189	-0.173	-.508**	-.465**	-.524**
**DTA**	.282*	.302*	.790**	.813**	1	-0.186	-0.045	-0.097	-0.207	-0.096	-0.128	-0.170	-0.147	-0.109	0.060	-0.163	0.248	0.004	0.134	-0.192	-.344**	-.356**	-.393**
**Ll_Head**	-0.173	0.221	-.341**	-.332**	-0.186	1	.624**	.863**	.969**	.685**	.857**	.557**	.268*	.321*	0.235	.526**	.334**	.494**	-0.242	0.117	.294*	0.239	.286*
**Lw_Head**	-0.100	.349**	-0.248	-0.247	-0.045	.624**	1	.921**	.609**	.948**	.884**	.659**	.309*	.356**	0.198	.646**	.466**	.609**	-0.236	0.004	.439**	.351**	.389**
**fLA_Head**	-0.128	.345**	-.294*	-.286*	-0.097	.863**	.921**	1	.846**	.918**	.973**	.659**	.271*	.347**	0.248	.646**	.461**	.609**	-0.221	0.083	.416**	.337**	.372**
**Ll_Anth**	-0.119	0.238	-.337**	-.325*	-0.207	.969**	.609**	.846**	1	.677**	.869**	.521**	0.192	.282*	0.229	.493**	.335**	.456**	-0.204	0.152	.274*	0.220	.259*
**Lw_Anth**	-0.081	.302*	-.299*	-.289*	-0.096	.685**	.948**	.918**	.677**	1	.944**	.641**	.292*	.358**	0.190	.628**	.467**	.575**	-.276*	0.132	.425**	.356**	.407**
**fLA_Anth**	-0.095	.313*	-.316*	-.303*	-0.128	.857**	.884**	.973**	.869**	.944**	1	.624**	0.237	.333**	0.233	.613**	.453**	.564**	-0.231	0.164	.395**	.329*	.370**
**S/Spike**	-0.238	0.177	-.384**	-.371**	-0.170	.557**	.659**	.659**	.521**	.641**	.624**	1	.571**	.615**	.344**	.961**	.574**	.883**	-.305*	0.181	.526**	.484**	.537**
**PH**	-.397**	-0.075	-0.191	-0.182	-0.147	.268*	.309*	.271*	0.192	.292*	0.237	.571**	1	.763**	.302*	.624**	.393**	.572**	0.032	0.002	.344**	.348**	.313*
SpL	-0.206	-0.093	-0.173	-0.173	-0.109	.321*	.356**	.347**	.282*	.358**	.333**	.615**	.763**	1	0.214	.662**	.444**	.605**	0.038	-0.029	.324*	.297*	.275*
T/P	-0.051	-0.011	-0.058	-0.026	0.060	0.235	0.198	0.248	0.229	0.190	0.233	.344**	.302*	0.214	1	.389**	.484**	.408**	0.153	.343**	0.226	0.220	0.163

Sp/S	-0.218	0.204	-.374**	-.354**	-0.163	.526**	.646**	.646**	.493**	.628**	.613**	.961**	.624**	.662**	.389**	1	.644**	.908**	-0.058	0.218	.581**	.537**	.531**
BY	0.013	0.168	0.160	0.171	0.248	.334**	.466**	.461**	.335**	.467**	.453**	.574**	.393**	.444**	.484**	.644**	1	.642**	0.123	.267*	0.244	0.211	0.159
GY	-0.203	0.243	-.289*	-.263*	0.004	.494**	.609**	.609**	.456**	.575**	.564**	.883**	.572**	.605**	.408**	.908**	.642**	1	-0.023	0.173	.530**	.478**	.474**
TKW	0.181	0.027	0.190	0.189	0.134	-0.242	-0.236	-0.221	-0.204	-.276*	-0.231	-.305*	0.032	0.038	0.153	-0.058	0.123	-0.023	1	0.097	0.049	0.042	-0.187
Chl_Till	0.092	0.091	-0.223	-0.173	-0.192	0.117	0.004	0.083	0.152	0.132	0.164	0.181	0.002	-0.029	.343**	0.218	.267*	0.173	0.097	1	.356**	.352**	.305*
Chl_Boot	-.319*	0.153	-.554**	-.508**	-.344**	.294*	.439**	.416**	.274*	.425**	.395**	.526**	.344**	.324*	0.226	.581**	0.244	.530**	0.049	.356**	1	.939**	.897**
Chl_Head	-.369**	0.046	-.524**	-.465**	-.356**	0.239	.351**	.337**	0.220	.356**	.329*	.484**	.348**	.297*	0.220	.537**	0.211	.478**	0.042	.352**	.939**	1	.962**
Chl_Anth	-.413**	0.015	-.586**	-.524**	-.393**	.286*	.389**	.372**	.259*	.407**	.370**	.537**	.313*	.275*	0.163	.531**	0.159	.474**	-0.187	.305*	.897**	.962**	1

**. Correlation is significant at the 0.01 level (2-tailed).

*. Correlation is significant at the 0.05 level (2-tailed).

Table S2: Pearson correlation coefficients among antioxidant enzymes, photosynthetic pigments, and oxidative stress markers in wheat cultivars under nickel stress. *Significant positive and negative correlations highlight the relationships between POD, SOD, CAT, carotenoids, chlorophyll components (Chl.a, Chl.b, and Total Chl), MDA, and H_2O_2, illustrating the biochemical and physiological interactions that contribute to stress response. Significant correlations are denoted at the 0.01 (**) and 0.05 (*) levels.*

	POD	SOD	CAT	Carotenoid	Chl.a	Chl.b	Total Chl	MDA	H_2O_2
**POD**	1	0.226	0.002	.278*	.292*	.278*	.359**	-0.068	-.324*
**SOD**	0.226	1	0.016	.558**	.408**	.669**	.612**	-0.137	0.085
**CAT**	0.002	0.016	1	0.093	0.088	0.036	0.062	0.044	-0.055
**Carotenoid**	.278*	.558**	0.093	1	.884**	.801**	.956**	0.108	-0.043
**Chl.a**	.292*	.408**	0.088	.884**	1	.519**	.858**	-0.079	-0.022
**Chl.b**	.278*	.669**	0.036	.801**	.519**	1	.880**	0.028	0.000
**Total Chl**	.359**	.612**	0.062	.956**	.858**	.880**	1	-0.020	-0.007
**MDA**	-0.068	-0.137	0.044	0.108	-0.079	0.028	-0.020	1	.320*
**H_2O_2**	-.324*	0.085	-0.055	-0.043	-0.022	0.000	-0.007	.320*	1

*. Correlation is significant at the 0.05 level (2-tailed).

**. Correlation is significant at the 0.01 level (2-tailed).

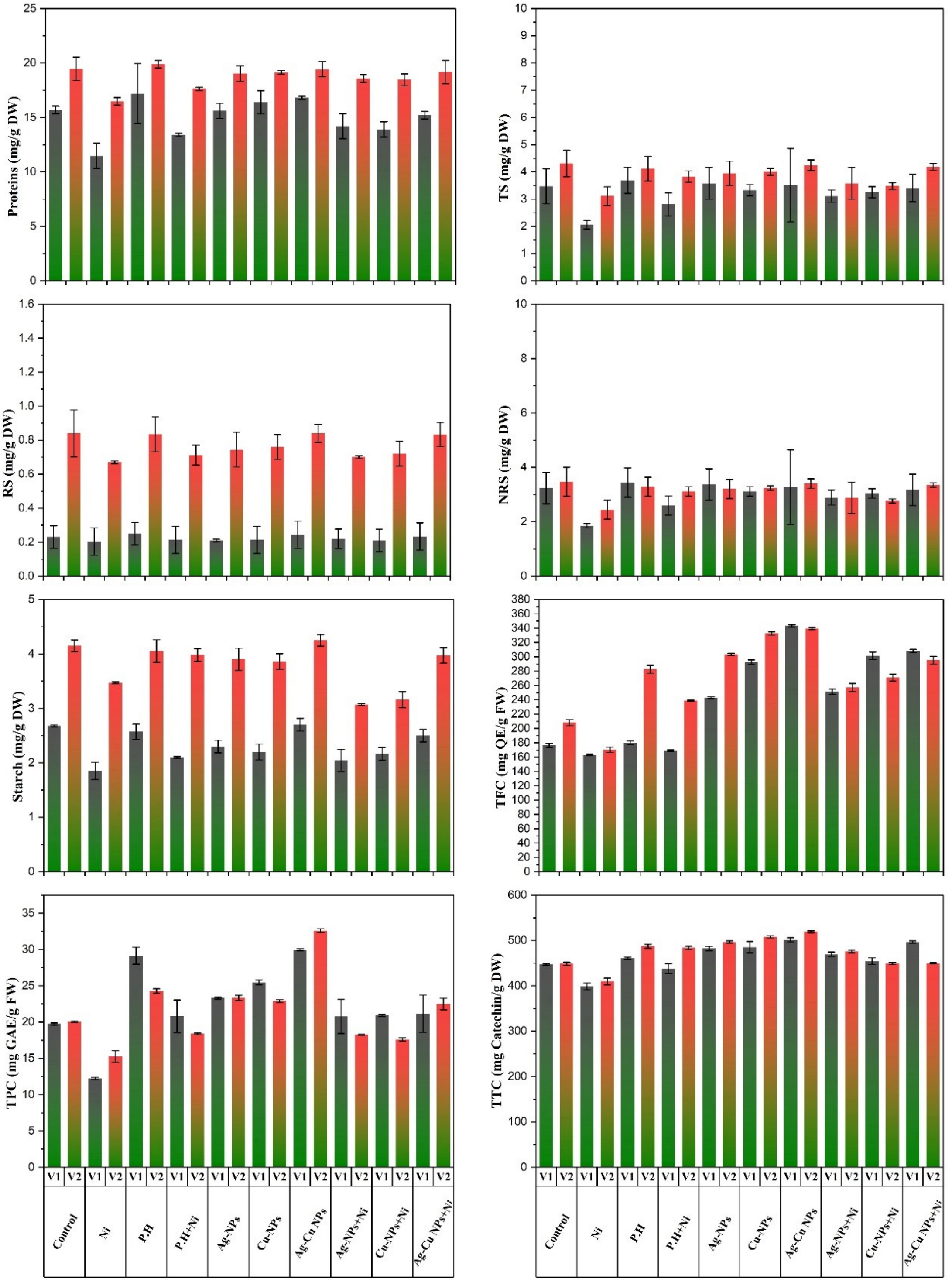

Figure S1: Phytochemical and nutritional content in seeds of wheat varieties (V1 and V2) under various treatments. *Parameters include proteins (mg/g DW), total sugars (TS; mg/g DW), reducing sugars (RS; mg/g DW), non-reducing sugars (NRS; mg/g DW), starch (mg/g DW), total flavonoid content (TFC; mg QE/g FW), total phenolic content (TPC; mg GAE/g FW), and total tannin content (TTC; mg TAE/g FW). Treatments with Ag and Cu nanoparticles,*

particularly Ag-Cu NPs+Ni, showed enhanced phytochemical content, indicating improved antioxidant responses and nutritional quality under stress. Bars represent mean values ± SE.
